# Kinetic Analysis of Cyclization by the Substrate-Tolerant Lanthipeptide Synthetase ProcM

**DOI:** 10.1021/acscatal.4c06216

**Published:** 2024-11-27

**Authors:** Emily K. Desormeaux, Garrett J. Barksdale, Wilfred A. van der Donk

**Affiliations:** † Department of Chemistry, University of Illinois at Urbana−Champaign, 600 South Mathews Avenue, Urbana, Illinois 61801, United States; ‡ School of Molecular and Cellular Biology, University of Illinois at Urbana−Champaign, 600 South Mathews Avenue, Urbana, Illinois 61801, United States; § Howard Hughes Medical Institute, University of Illinois at Urbana−Champaign, 600 South Mathews Avenue, Urbana, Illinois 61801, United States

**Keywords:** macrocycle, peptide cyclization, RiPPs, macrocyclic library, diversity generation

## Abstract

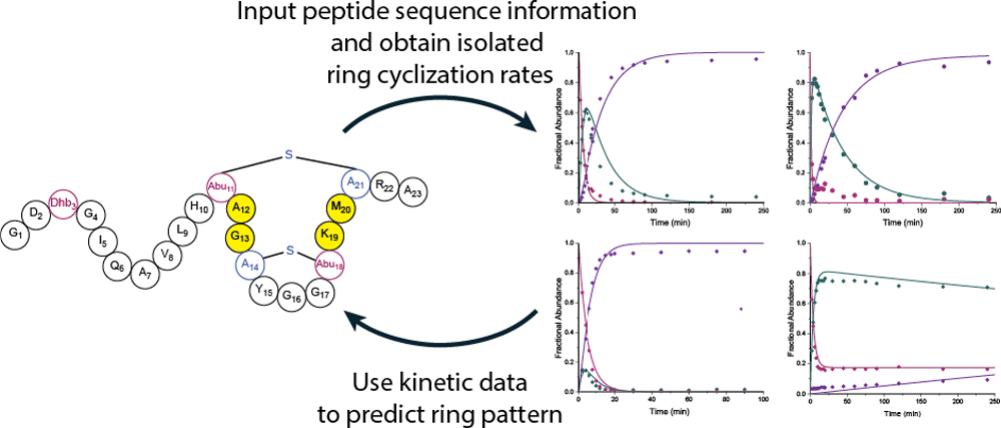

Lanthipeptides are ribosomally synthesized and post-translationally modified peptides (RiPPs) characterized by the presence of thioether cross-links called lanthionine and methyllanthionine, formed by dehydration of Ser/Thr residues and Michael-type addition of Cys side chains onto the resulting dehydroamino acids. Class II lanthipeptide synthetases are bifunctional enzymes responsible for both steps, thus generating macrocyclic natural products. ProcM is part of a group of class II lanthipeptide synthetases that are known for their remarkable substrate tolerance, having large numbers of natural substrates with highly diverse peptide sequences. They install multiple (methyl)lanthionine rings with high accuracy, attributes that have been used to make large libraries of polycyclic peptides. Previous studies suggested that the final ring pattern of the lanthipeptide product may be determined by the substrate sequence rather than by ProcM. The current investigation on the ProcM-catalyzed modification of one of its 30 natural substrates (ProcA3.3) and its sequence variants utilizes kinetic assays to understand the factors that determine the ring pattern. The data show that changes in the substrate sequence result in changes to the reaction rates of ring formation that in some cases lead to a change in the order of the modifications and thereby bring about different ring patterns. These observations provide further support that the substrate sequence determines to a large degree the final ring pattern. The data also show that similar to a previous study on another substrate (ProcA2.8), the reaction rates of successive reactions slow down as the peptide is matured; rate constants observed for the reactions of these two substrates are similar, suggesting that they reflect the intrinsic activity of the enzyme with its 30 natural substrates. We also investigated whether rates of formation of single isolated rings can predict the final ring pattern of polycyclic products, an important question for the products of genome mining exercises, as well as library generation. Collectively, the findings in this study indicate that the rates of isolated modifications can be used for predicting the final ProcM-produced ring pattern, but they also revealed limitations. One unexpected observation was that even changing Ser to Thr and vice versa, a common means to convert lanthionine to methyllanthionine and vice versa, can result in a change in the ring pattern.

## Introduction

**Figure 1:**
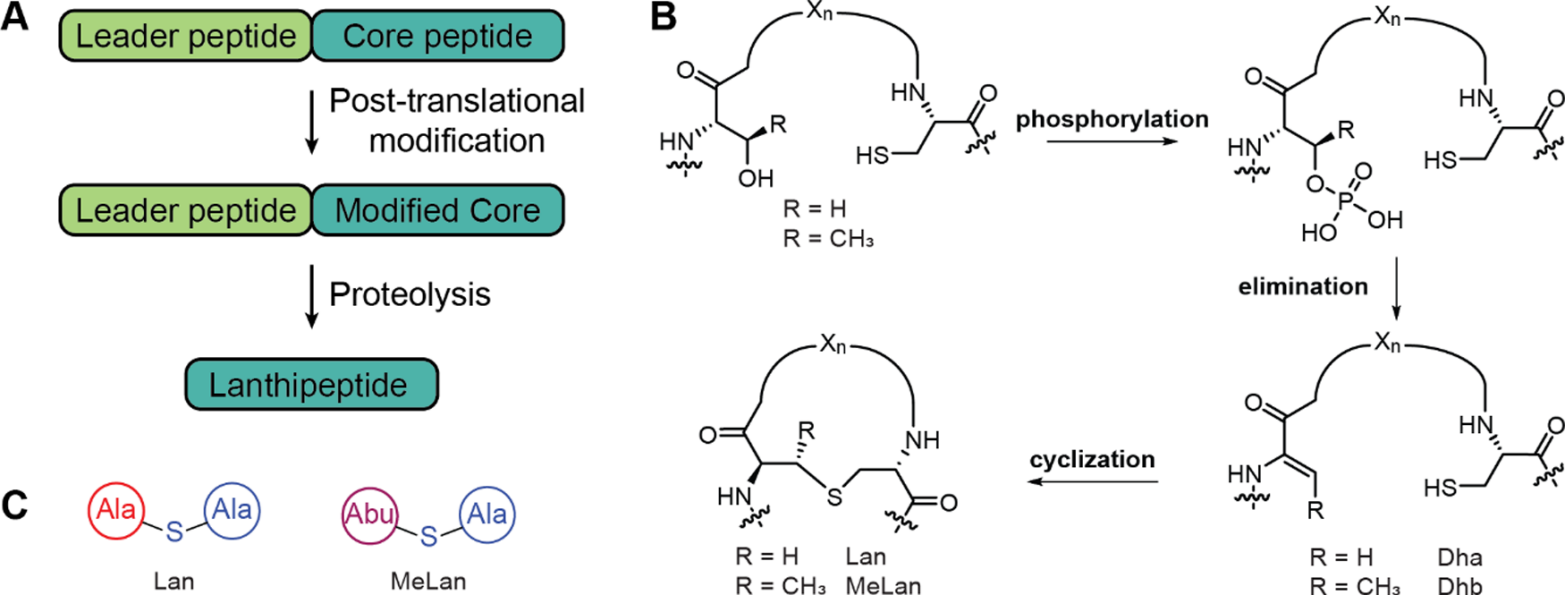
Biosynthetic pathways to lanthipeptides. (A) Generic scheme for lanthipeptide biosynthesis. (B) Biosynthetic scheme for the post-translational modifications installed by class II lanthipeptide synthetases. (C) Shorthand notation for lanthionine (Lan) and methyllanthionine (MeLan).

Natural products are an abundant source of compounds with diverse bioactivities. Approximately two-thirds of all drugs approved between 1981 and 2019 are natural products or their derivatives.^1^ Ribosomally synthesized and post-translationally modified peptides (RiPPs) are a class of natural products that exhibit a wide array of biological activities.^2^ Naturally occurring RiPPs have recently emerged as an exciting starting point to make large libraries of macrocyclic peptides that can be screened or selected for novel activities.^3−9^ The biosynthetic gene clusters (BGCs) for these compounds contain a gene encoding a precursor peptide that is post-translationally modified by biosynthetic enzymes and proteolytically cleaved to remove a leader peptide and produce the mature natural product (e.g., Figure 1A).^10−12^ The precursor peptide is composed of an N-terminal leader region, which is typically important for enzyme recognition, and a C-terminal core region, which undergoes modification. Many post-translational modification enzymes have proven tolerant of changes in the core peptide sequence as long as the leader sequence is present to guide the enzymes.^11^ The factors that make these enzymes so forgiving in their catalytic activity yet result in single macrocyclic products are still poorly understood.

Lanthipeptides are a class of RiPPs characterized by the presence of lanthionine (Lan) and/or methyllanthionine (MeLan).^10^ These thioether cross-links are installed by (at least) five different lanthipeptide synthetase classes through the dehydration of Ser/Thr residues and addition of a Cys to the dehydrated residue.^10,11,13−19^ Class II lanthipeptide synthetases, LanMs, are bifunctional enzymes that catalyze both the dehydration and cyclization reactions (Figure 1B). The N-terminal dehydratase domain catalyzes the dehydration of Ser/Thr residues using ATP to first generate a phosphorylated peptide intermediate, which undergoes elimination in the same active site to yield dehydroalanine or dehydrobutyrine (Dha and Dhb, respectively).^20,21^ The C-terminal cyclization domain catalyzes the Michael-type addition of a Cys thiol to a dehydrated residue to produce the (Me)Lan ring(s).^22^ A growing subset of class II lanthipeptide synthetases are found alongside a large number of precursor peptides (LanAs; 10–80)^23−27^ deviating from the majority of RiPP BGCs, which typically encode a single precursor gene. These gene clusters have been of particular interest for bioengineering efforts since the proteins have demonstrated relaxed substrate specificity and they have been used to make libraries of macrocyclic peptides,^3,4,26,28−36^ a new drug modality that is attracting increasing interest in academia and industry.^37−41^

ProcM is a highly substrate-tolerant class II lanthipeptide synthetase discovered in the marine picocyanobacterium *Prochlorococcus* MIT9313.^23^ Along with the gene encoding ProcM, 30 putative genetically encoded precursor peptides, termed ProcAs, were identified within the genome.^23,28^ Of these precursors, 18 ProcA peptides have been tested and were all modified by ProcM to produce polycyclic products with defined ring patterns termed prochlorosins (Pcns).^23,42,43^ The extreme substrate tolerance of ProcM and orthologs like SyncM^26,33^ have prompted mechanistic studies to try and understand how the enzymes can combine high accuracy of the rings produced with high tolerance for the sequence of the precursor peptide. Mechanistic studies with ProcM support a kinetically controlled mechanism, as the retro-Michael addition was not observed.^44,45^ Kinetic analysis performed on ProcM-catalyzed modification of the substrate ProcA2.8 supports this hypothesis and suggests that the rates of each ProcM-catalyzed cyclization progressively decrease as the peptide acquires successive modifications.^46^

ProcM and similar substrate-tolerant LanM enzymes were initially identified in *Prochlorococcus* and *Synechococcus* strains.^23,25,26^ A recent deep dive into 47 *Prochlorococcus* and *Synechococcus* genomes increased the number of LanAs identified within these marine bacteria, with 484 putative peptides identified compared to the previously reported 181. The study also identified more putative ProcA peptides within the *Prochlorococcus* MIT9313 genome, suggesting the organism could have as many as 40 precursor peptides.^27^ All of the LanMs identified in this analysis were determined to be ProcM-like (which have a unique set of amino acid ligands to the catalytic Zn^2+^ in the cyclase domain^24^). Phylogenetic analysis also showed that the ProcM-like enzymes are in many other cyanobacteria.^24^ Hence, a better understanding of catalysis by ProcM would inform on these systems that have much potential for engineering.

**Figure 2:**
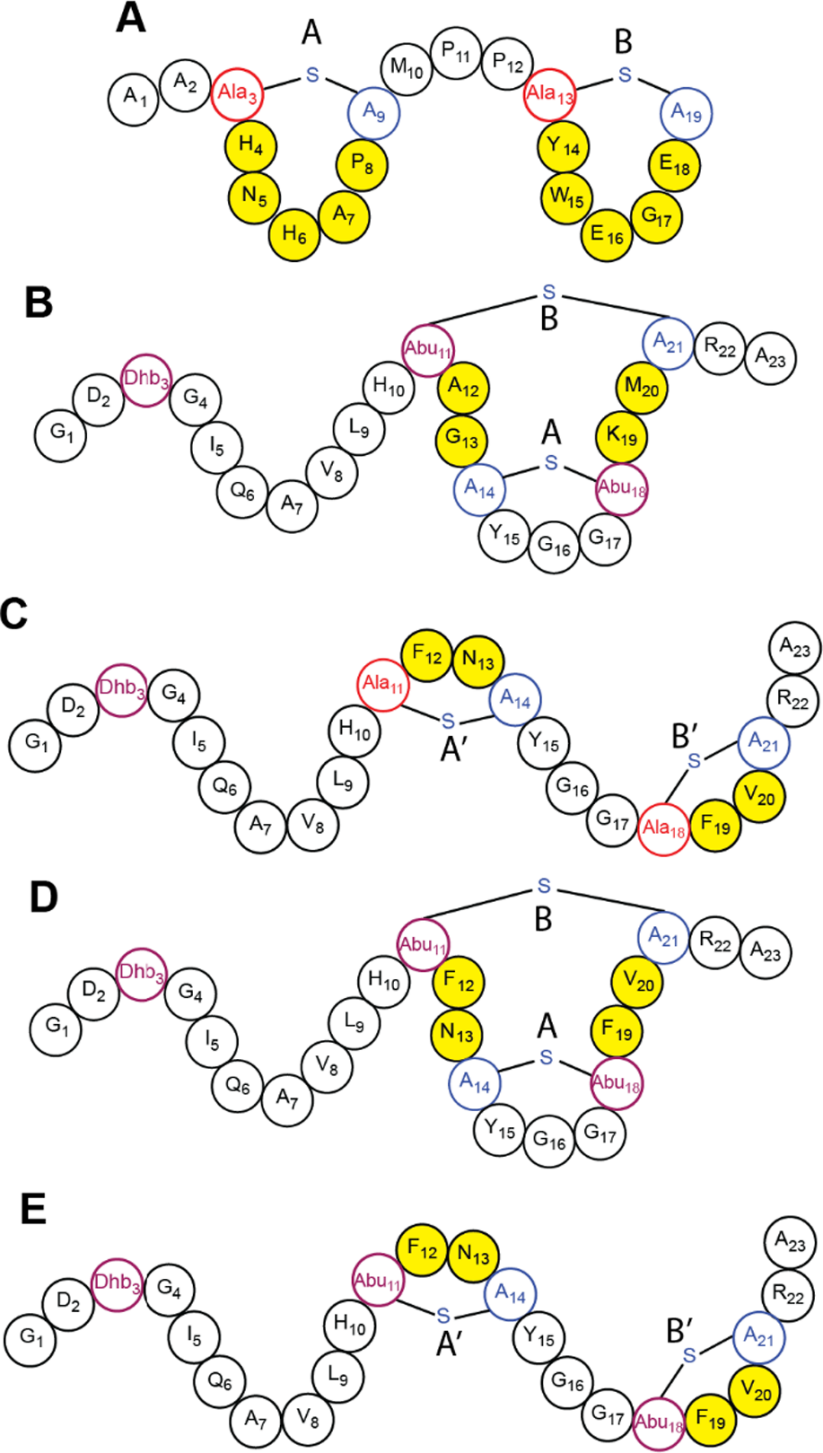
Structure of prochlorosin peptides represented by shorthand notation. (A) Pcn2.8. (B) Pcn3.3 WT with an overlapping ring pattern containing A and B rings. (C) Pcn3.3 variant Z with a nonoverlapping ring pattern containing A’ and B’ rings. (D, E) MeLan-containing Pcn3.3 variant Z, which produces a mixture of the overlapping and nonoverlapping ring patterns. The positions that were randomized in the ProcA2.8 and ProcA3.3 analogue libraries are indicated in yellow.

The ability of ProcM to produce Pcns with a wide diversity of ring patterns has already resulted in the utilization of ProcM for bioengineering efforts.^3,4,29−31,47^ Tested members from a ProcA2.8 analogue library (Figure 2A) all formed the canonical ring pattern present in the wild-type (WT) peptide, whereas a library composed of ProcA3.3 variants produced a mixture of the native overlapping and non-native nonoverlapping ring patterns (Figure 2).^4,31^ These findings support a model in which the final Pcn ring pattern is dictated by the precursor peptide sequence, which may be driven by conformational preferences.^48^ Cyclization of dehydrated ProcA3.3 also results in the non-native nonoverlapping pattern in the absence of ProcM,^45^ similar to the predictions of computational models.^48^ These results suggest that while precursor peptide sequence is an important factor, ProcM also plays some role in governing the final ring pattern.^49^ To determine the importance of substrate sequence and ProcM influence on Pcn maturation, kinetic analysis was utilized in this work to characterize the maturation of WT ProcA3.3 with an overlapping ring pattern (Figure 2B) as well as a ProcA3.3 variant (variant Z) that forms a nonoverlapping ring pattern (Figure 2C).

In addition to these analyses, the importance of the dehydratable residue identity (to form MeLan versus Lan) and the cyclization rates for individual rings were evaluated to determine the factors governing the final ring pattern. Unanticipated results were observed, such as a change in ring pattern upon substituting MeLan by Lan cross-links. Measurement of the cyclization kinetics for individual rings resulted in a trend that allowed for the prediction of the ring pattern of the fully modified parent peptide, but the findings of this study also suggest that prediction of ring patterns may be challenging for variants of the native substrates of diversity-generating lanthipeptide synthetases like ProcM.

## Methods

### ProcM Kinetic Assays

ProcA peptides and ProcM were expressed and purified as previously described.^46,48^ ProcM (2 or 4 μM) and ProcA peptide (80, 120, or 160 μM) were preincubated separately at 25 °C for 1 h in 750 μL of the reaction mixture (5 mM ATP, 0.17 mM ADP, 5 mM MgCl_2_, 100 mM HEPES, 0.1 mM TCEP, pH 7.5) to allow for equilibration prior to initiation of the reaction. The reaction mixture was altered from previous methods with the addition of ADP.^46^ ADP was added to the reaction mixture as it helps to limit buildup of phosphorylated intermediates and allows intermediates to rebind ProcM when present; these intermediates, when observed in previous publications, often act as dead-end products in the absence of ADP that are not converted to the dehydrated intermediate.^46^ Under physiological conditions, ADP would be present in the ATP/ADP ratio used here.^50^ The peptide and protein samples were mixed thoroughly to initiate a reaction. At the desired time points (2, 4, 6, 8, 10, 15, 18, 20, 30, 45, 60, 75, 90, 120, 180, and 240 min), 80 μL aliquots were removed from the reaction. The reaction aliquot was quenched using 900 μL of quench buffer (111 mM citrate and 1.11 mM EDTA, pH 3.0) and placed on ice until completion of all time points. Quenched samples were reduced with the addition of 100 μL of 100 mM TCEP, and the samples were incubated at 25 °C for 10 min. The pH was then adjusted to approximately 6.3 with the addition of 40–45 μL of 5 M NaOH. Noncyclized cysteine residues were then alkylated by the addition of 11 μL of 1 M *N*-ethylmaleimide (NEM) in EtOH, and the alkylation reaction was allowed to proceed for 10 min at 37 °C before quenching with the addition of 11 μL of formic acid. The solvent for each sample was then exchanged with water via centrifugal filtration using Amicon 3 kDa filters. The centrifugation step was altered from published protocols due to the discontinuation of Vydac BioSelect columns.^46^ Samples were lyophilized and stored at −80 °C until analysis.

### Liquid Chromatography-Mass Spectrometry

Dried samples were resuspended in 50 μL of water, and the total peptide concentration was estimated via UV–vis absorption at 280 nm. Samples were then diluted to 15 μM total peptide in LCMS solvent (50% ACN in H_2_O, 0.1% formic acid). Samples (10 μL) were injected into an Acquity UPLC BEH C8 1.7 μm column (1.0 × 100 mm) coupled to a Waters quadrupole/time-of-flight (Q/ToF) Synapt-G1 series mass spectrometer. The samples were analyzed with a flow rate of 0.4 mL/min, and the peptides were eluted using the following protocol: 3% B held for 2 min, 3–97% B over 3 min, and held at 97% B for 3 min followed by re-equilibration of the column to starting conditions (A, 0.1% formic acid in water; B, 100% ACN, 0.1% formic acid). All of the peptides eluted between 5 and 6 min in a single broad peak. All samples were run in a randomized order, and 10 μL injections of water were run between each sample to prevent cross contamination. The mass spectrometry conditions were as follows: positive ion mode, V optics, capillary voltage = 3.0 kV, cone gas = 180 L/h, desolvation gas = 600 L/h, source temperature = 120 °C, desolvation temperature = 200 °C. The detector was set to a *m*/*z* window of 800–1800 Da with a 1 s scan time. External calibration was performed by using a 0.1% phosphoric acid standard. The identity of each ion was determined by HRMS (Table S1) and MS/MS fragmentation patterns.

The total ion chromatogram (TIC) for each sample was used to determine the *m*/*z* values for ions of interest. These *m*/*z* values were then used along with a mass window around the center of the most intense isotopic peak to generate extracted ion chromatograms (EICs). Integration of the EICs using Waters MassLynx software was performed, and the peak areas were then used to calculate the fractional abundance of each peptide species using previously published methods.^46^ All peptides showed a linear response with respect to concentration in the LCMS analysis (Figure S1G). As previously discussed, the ionization efficiencies of all peptides are very similar because they all contain the same 67-amino acid leader peptide (Figure S1H).

### Simulation of Kinetic Data

The calculated time-dependent fractional abundances for each sample were imported into KinTek Explorer,^51,52^ and the standard deviation for each ion during the time course was estimated via fitting to exponential equations using the “aFit” module. A kinetic model was then designed in KinTek, and the time courses were used to fit the model to the collected data. Simplifying assumptions were used to create a minimal kinetic model that described the data collected, following previously published methods.^46^ Binding constants of ProcA peptides to ProcM from previous studies were used to approximate binding for all of the peptides utilized in this study. Goodness-of-fit of the models was evaluated within the KinTek software by χ^2^/DoF (the degrees of freedom), wherein for a good fit, χ^2^/DoF approaches 1. DoF is defined as *n* – 1, where *n* is the number of variable parameters in the designed kinetic model (χ^2^/DoF plots are shown in the Supporting Information).

The FitSpace Explorer module of KinTek was then utilized to generate confidence contours and evaluate the quality of the data fitting. A confidence contour is a plot of the χ^2^_min_/χ^2^ value as a function of a variable parameter and can provide information on how well constrained that parameter is by the given model as well as providing well-defined upper and lower confidence intervals. This type of analysis is helpful when the distribution of errors is uneven (i.e., well-defined lower boundary and poorly defined upper boundary). The parameter boundaries for the rates in the kinetic models for WT ProcA3.3 and ProcA3.3 variant Z and their single ring analogues were calculated using χ^2^ thresholds of 0.83 and 0.96, respectively (see Supporting Information), which were the recommended boundaries by FitSpace Explorer.

### Liquid Chromatography Mass Spectrometry–Fragmentation analysis

To determine the location of modifications in partially modified ProcA3.3 variant Z, the remaining sample after kinetic analysis of the 10 min time point of the 80 μM kinetic reaction was analyzed using previously published methods.^48^ To determine the final ring pattern of fully modified ProcA3.3 variants, the remaining sample after kinetic analysis of the end point of the 40 μM kinetic reaction was analyzed using previously published methods.^48^

## Results and Discussion

### Modification of ProcA3.3 WT by ProcM

**Figure 3:**
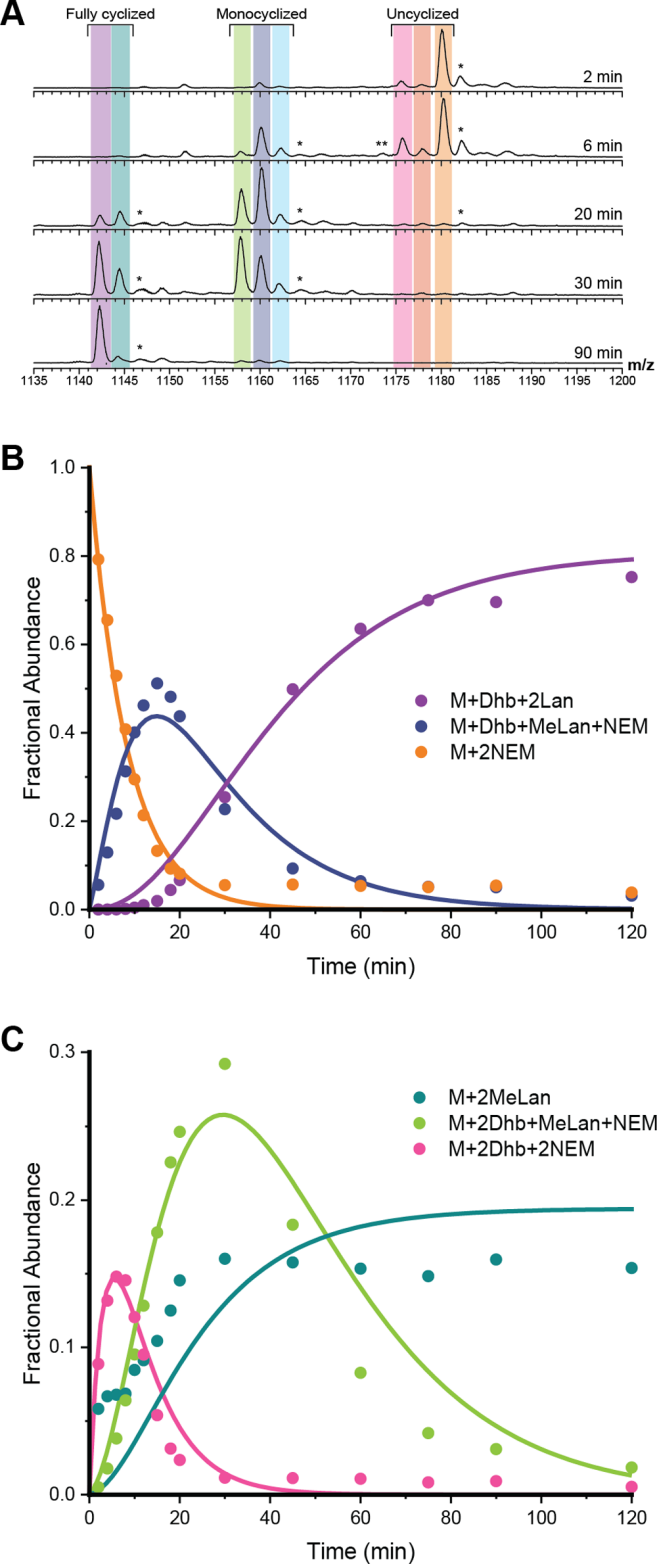
ProcA3.3 WT kinetic analysis. (A) Representative ESI mass spectra of the 8+ charge state of ProcA3.3 WT at multiple time points. For each trace, the *y*-axis is scaled to the intensity of the highest peak present. The sample was treated with NEM to investigate the cyclization state of the Cys residues. Coloring of each ion: light orange, unmodified peptide (M + 2 NEM); dark orange, once dehydrated peptide (M + Dhb + 2 NEM); pink, twice dehydrated peptide (M + 2 Dhb + 2 NEM); light blue, once dehydrated and cyclized peptide (M + MeLan + 1 NEM); dark blue, twice dehydrated and once cyclized peptide (M + Dhb + MeLan + 1 NEM); green, thrice dehydrated and once cyclized peptide (M + 2 Dhb + MeLan + 1 NEM); teal, twice dehydrated and twice cyclized peptide (M + 2 MeLan); purple, thrice dehydrated and twice cyclized peptide (M + Dhb + MeLan). (B) Time course showing unmodified peptide (orange), peptide containing one MeLan ring and one additional dehydration (dark blue), and the final product (purple; M–3 H_2_O). (C) Time course for minor observed intermediates including noncyclized peptide with two dehydrations (pink), peptide containing one MeLan ring with two dehydrations (green), and peptide containing two MeLan rings with no additional dehydrations (teal). Reaction for all panels was performed with 60 μM ProcA3.3 WT and 2 μM ProcM. Peaks labeled with an asterisk (*) are oxidation products that were present in the starting peptide (e.g., Figure S12C,D) and that were carried through to oxidized products. They were omitted from the modeling but when included did not substantially change the calculated rate constants. Peaks labeled with two asterisks (**) are triply dehydrated starting peptides that did not cyclize and that were also not included in the model.

The maturation of ProcA3.3 WT (Figure 2B) involves the dehydration of three residues, Thr3, Thr11, and Thr18 as well as the cyclization of two overlapping MeLan rings, the A ring (formed between Cys14 and Thr18) and the B ring (formed between Thr11 and Cys21). A previous study showed that the A ring forms before the B ring,^44^ but the timing of dehydration compared to cyclization was not determined. For the current kinetic analysis, the reaction mixture was subjected to NEM alkylation of unreacted Cys residues at various time points, resulting in uncyclized intermediates that are differentiated from cyclized peptides via a mass shift. Relevant peptides were then monitored via LC-MS analysis, and fractional abundances were used to monitor the appearance and consumption of peptides (Figure 3).^46^

The reaction with WT ProcA3.3 was complete after approximately 45 min when only 3-fold dehydrated and twice cyclized peptide (purple) and 2-fold dehydrated and twice cyclized peptide (teal) were detected (Figure 3A). The main intermediates observed are 2- and 3-fold dehydrated peptides that have a single ring (dark blue and light green) and twice dehydrated peptide with no rings (pink). Intermediates containing a single dehydration (dark orange) and peptide containing a single methyllanthionine (light blue) were observed in low abundance (Figure 3A). These intermediates were not used for the final minimal kinetic model. The peptide intermediate containing two MeLan rings and lacking the third dehydration (teal) was modeled as an end product, as this species remained through the reaction (Figure 3C, teal). We hypothesize that the twice cyclized product is unable to reach the dehydration active site of ProcM.

**Scheme 1:**
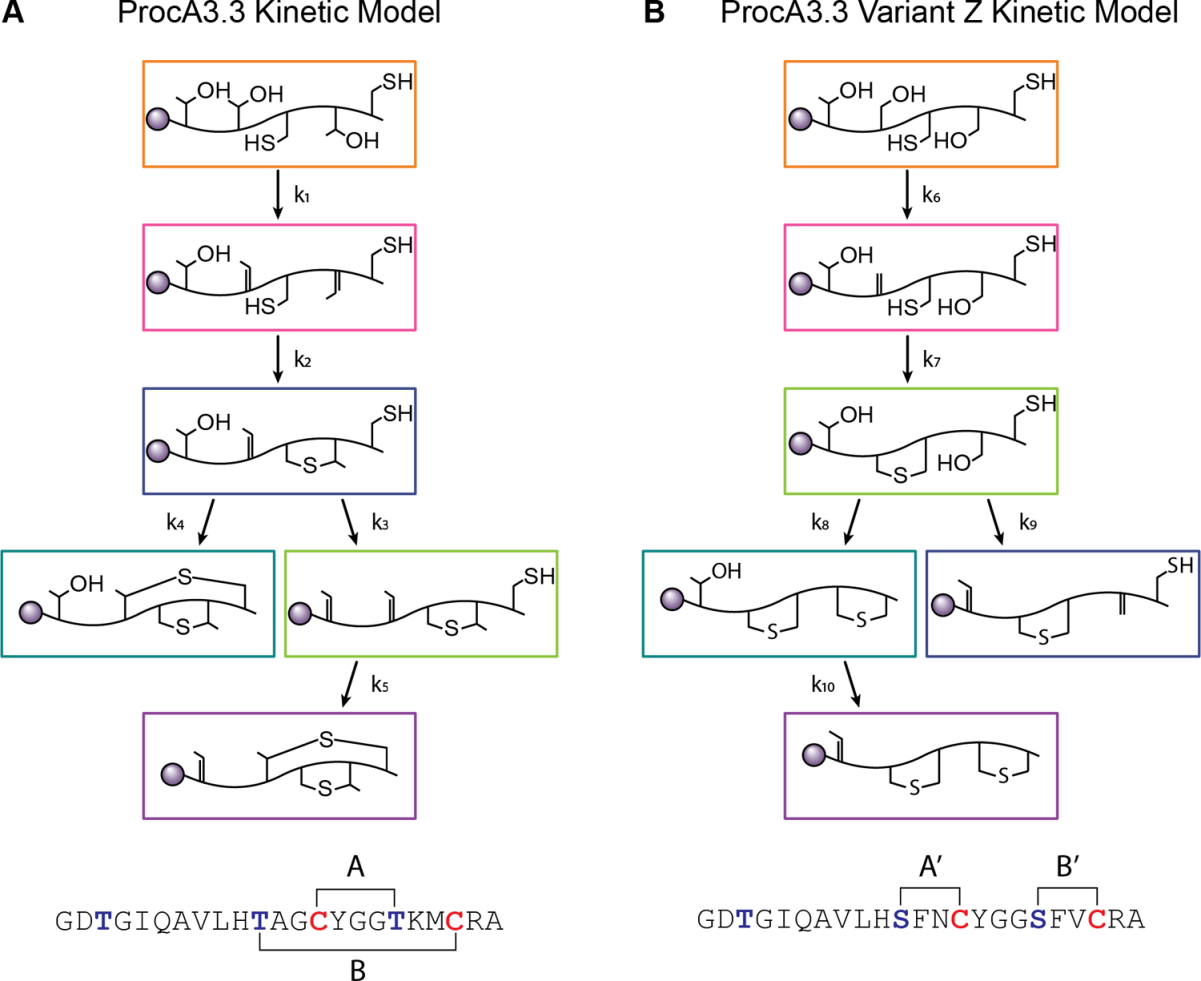
Kinetic Models for WT ProcA3.3 and Variant Z (A) Proposed minimal kinetic model for ProcA3.3 WT. Reactions for the initial two dehydrations were combined because the singly dehydrated intermediate is not observed in significant quantities under the reaction conditions. Color coding as in Figure 3. (B) Proposed minimal kinetic model for ProcA3.3 variant Z. Notable differences are installation of a lanthionine ring before the second and third dehydrations occur. The sequences for both peptides are shown below their respective kinetic model. All peptides contain an N-terminal 67-amino acid leader peptide (Figure S1H) that is depicted as a gray circle.

A minimal kinetic model representing the processing of WT ProcA3.3 by ProcM is depicted in Scheme 1A. Rate constants were determined for each chemical step via simulation of the data in Figure 3 using KinTek Explorer as described in the Methods section. Several simplifying assumptions were used for the creation of this minimal model as described previously.^46^ Binding constants obtained in a previous study for one of the substrates were used for all of the peptides and intermediates. This assumption is based on the presence of the same long leader region for all ProcA3.3 substrates and intermediates (Figure S1H) and the major contribution of the leader peptide to the binding affinity for ProcM. Additionally, all chemical steps are assumed and modeled as irreversible. These assumptions are supported by dehydration being driven by ATP hydrolysis and ProcM having been demonstrated not to catalyze retro-Michael additions.^45^ To increase confidence, the model was used for a global fit of multiple experiments conducted with varying concentrations of substrate and enzyme (Figure S1). The binding and chemical rate constants thus obtained are provided in Table 1.

**Table 1:** Summary of the Simulated Rate Constants for Fully Modified Substrates

*k*_*x*_	modification	best fit ± SE (min^–1^)	FitSpace boundaries (min^–1^)^a^	*k*_net_ (μM^–1^ min^–1^)^b^
*k*_on_		4.26^c^^,^^d^	Held constant	
*k*_off_		20^d^	Held constant	
**ProcA3.3 WT**				
*k*_1_	Thr11 and Thr18 dehydration	4.8 ± 0.4	3.9–5.9	0.82
*k*_2_	A ring cyclization	14 ± 3	9–25	1.8
*k*_3_	Thr3 dehydration	1.7 ± 0.2	1.3–2	0.33
*k*_4_	B ring cyclization	0.40 ± 0.06	0.27–0.56	0.1
*k*_5_	B ring cyclization	1.8 ± 0.2	1.3–2.6	0.35
**ProcA3.3 variant Z**				
*k*_6_	Ser11 dehydration	3.8 ± 0.3	3.4–4.3	0.68
*k*_7_	A’ ring cyclization	34 ± 17	21.2–166	2.7
*k*_8_	B’ ring formation (dehydration and cyclization)	16 ± 4	11–27	1.9
*k*_9_	Thr3 dehydration	1.4 ± 0.1	1.3–1.6	0.28

a FitSpace boundaries are reported for a χ^2^ threshold boundary of 0.83 for WT ProcA3.3 and 0.96 for ProcA3.3 variant Z (see Figures S1 and S3).

b *k*_net_= (*k*_on_*k*_*x*_)/(*k*_off_ + *k*_*x*_).

c *k*_on_ is in units of μM^–1^min^–1^.

d *k*_on_ and *k*_off_ values were used from previously published data.^46^

The rate constants in Table 1 are similar to those reported previously for modification of ProcA2.8 by ProcM. The first dehydration of ProcA2.8 occurs at a rate of 1.4 min^–1^ and the second dehydration at a rate of 14 min^–1^.^46^ For ProcA3.3, the first dehydration has a rate constant of 4.8 min^–1^ and the second dehydration is too fast to allow for its determination. The rate constants associated with cyclization, *k*_2_ for ring A (14 min^–1^) and *k*_4/5_ for ring B (0.4 and 1.8 min^–1^), also follow a similar trend to that of ProcA2.8,^46^ in which the first cyclization reaction (2 min^–1^) was also considerably faster than that of the second ring (0.28 min^–1^). *The rates previously observed for WT ProcA2.8 and recorded here for WT ProcA3.3 suggest that ProcM modifies its substrates with similar efficiencies despite the very different ring patterns involved.*

For ProcA3.3, the second cyclization reaction was modeled to occur from two possible intermediates; cyclization from a twice dehydrated intermediate (*k*_4_) appears to produce a product that cannot be dehydrated a third time, whereas cyclization from a three-times dehydrated peptide (*k*_5_) produces the fully modified product. The rate constants for these reactions were different (*k*_4_ = 0.40 min^–1^ and *k*_5_ = 1.8 min^–1^).

### ProcA3.3 Variant Z Modification by ProcM

The ProcA3.3 variant (Figure 2) used in this study was selected from a library of ProcA3.3 analogues made to disrupt protein–protein interactions.^4,31^ Library members contained two changes compared to WT. First, the analogues featured T11S and T18S mutations that result in the formation of two Lan rings rather than MeLan rings. Second, the members of the library were randomized at four positions (shown in yellow in Figure 2) to assess the effect on cyclization pattern. The library member chosen for kinetic analysis in this study (termed variant Z) was fully modified by ProcM and produced almost exclusively the nonoverlapping ring pattern. Under the reaction conditions provided in the Methods section, only intermediates corresponding to the nonoverlapping ring pattern were observed by tandem-MS analysis (Figure S2). Therefore, variant Z was a good candidate for kinetic analysis to understand the origins of the different cyclization selectivity compared to that of WT ProcA3.3.

**Figure 4:**
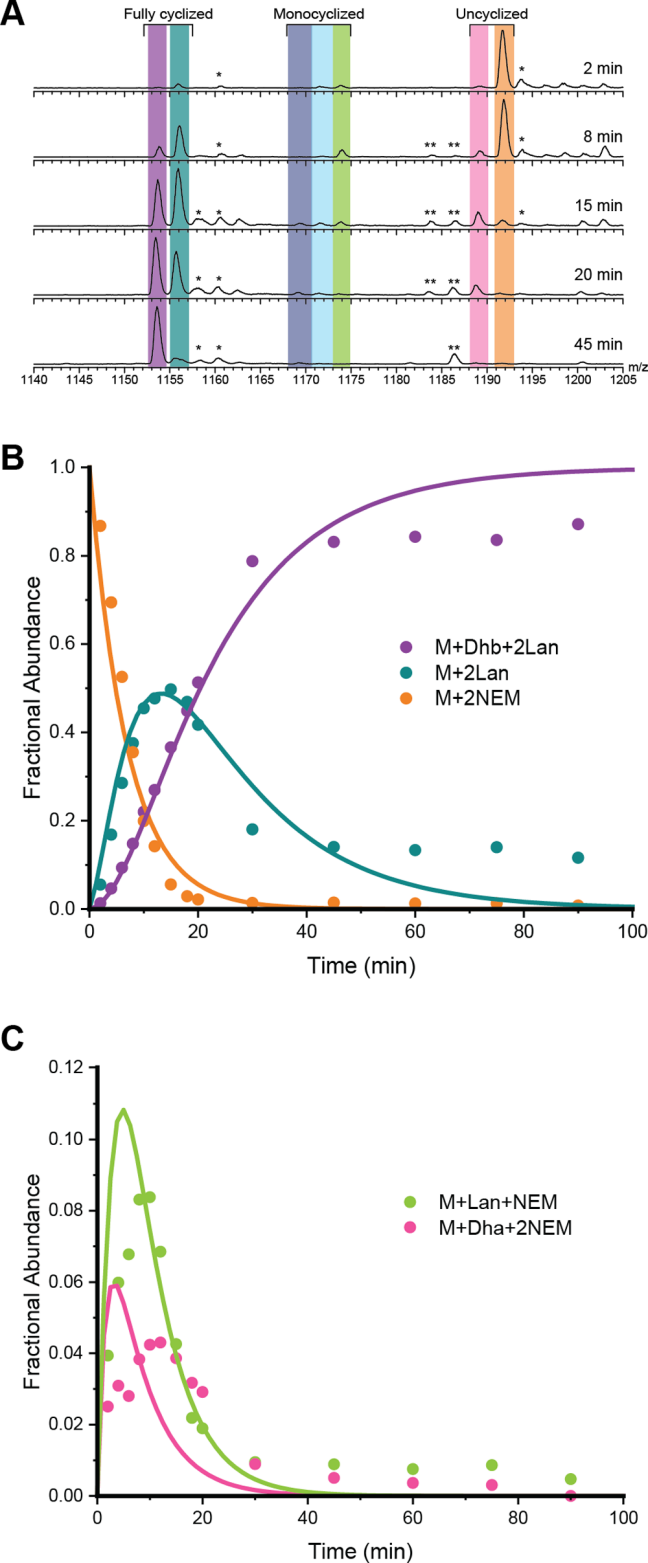
ProcA3.3 variant Z kinetic analysis. (A) Representative ESI mass spectra of the 8+ charge state of ProcA3.3 variant Z at multiple time points. Time course for the major species of the ProcA3.3 variant reaction with fractional abundances. Coloring of each ion: light orange, unmodified peptide (M + 2 NEM); pink, once dehydrated peptide (M + Dha + 2 NEM); green, once dehydrated and once cyclized peptide (M + Lan + 1 NEM); light blue, twice dehydrated and once cyclized peptide (M + Dha + Lan + 1 NEM); dark blue, thrice dehydrated and once cyclized peptide (M + 2 Dhx + Lan + 1 NEM); teal, twice dehydrated and twice cyclized peptide (M + 2 Lan + 1 NEM); purple, thrice dehydrated and twice cyclized peptide (M + Dhb + 2 Lan). (B) Time course for species including an unmodified peptide (orange), a peptide containing two Lan rings (cyan), and the final product (purple). (C) Time course for minor species including noncyclized peptide with one dehydration (pink) and peptide containing one Lan ring (green). Reaction conditions for all panels were 40 μM ProcA3.3 variant Z and 2 μM ProcM. Peaks labeled with an asterisk (*) are oxidation products that were present in the starting peptide and that were carried through to oxidized products (see Figure S12C,D). They were omitted from the modeling but when included did not substantially change the calculated rate constants. Peaks labeled with two asterisks (**) are doubly and triply dehydrated starting peptides that did not cyclize and that were also not included in the model.

To generate a minimal kinetic model for ProcA3.3 variant Z, intermediates were again monitored by LC-MS analysis (Figure 4), and tandem MS/MS analysis was utilized to determine the location of the modifications (e.g., Figure S2). Tandem MS/MS analysis with the singly cyclized intermediate present at the 10 min time point of a reaction containing 80 μM ProcA3.3 variant Z and 2 μM ProcM demonstrated that the NEM adduct was present on Cys21 and that dehydration occurred on Ser11, indicative of A’ ring formation (Figure S2). No ions were observed to suggest the presence of dehydration at other positions nor were NEM adducts detected at Cys14.

Two intermediates, one containing a single Lan ring and a single additional dehydration (light blue) and one containing a single Lan ring and two additional dehydrations (dark blue), were observed in low abundance (Figure 4A). These species accounted for only approximately four percent of the total peptide concentration at their highest abundance. These intermediates were not used in the final minimal kinetic model. The simplifying assumptions described above were again used to develop a model for variant Z. Rate constants were determined for each chemical step shown in Scheme 1B via simulation of data shown in Figures 4 and S3 using KinTek Explorer as described in the Methods section (Table 1).

The formation of the A’ ring indicates that the substitutions in the WT precursor sequence resulted in several changes in the order of modification of variant Z by ProcM. Most importantly, the A’ ring is formed prior to the second dehydration. Critically, this formation of the A’ ring sets the substrate on course to form a nonoverlapping ring pattern.

The dehydration rates of ProcA3.3 variant Z are comparable to those of ProcA3.3 WT. The first step in the kinetic model for each peptide, *k*_1_ representing the dehydration of both Thr18 and Thr11 in WT and *k*_6_ representing the dehydration of Ser11 in variant Z, has rates of 4.8 and 3.8 min^–1^, respectively. The final dehydration steps of Thr3, *k*_3_ for ProcA3.3 WT and *k*_9_ for ProcA3.3 variant Z, were 1.7 and 1.4 min^–1^, respectively. *The dehydration steps having comparable rate constants suggest that the presence or lack of the methyl group on the Ser vs Thr does not alter the rate of chemical modification for these substrates. This represents the first example of direct kinetic comparison of Ser/Thr dehydration at identical positions and in similar sequence contexts.*

The rate constants associated with cyclization for ProcA3.3 variant Z, *k*_7_ for the A’ ring and *k*_8_ for the B’ ring, were 34 and 16 min^–1^, respectively. The rate of B’ ring formation is a coupled rate constant of Ser18 dehydration and B’ ring formation. The absence of a dehydrated intermediate indicates that the cyclization rate is greater than that of dehydration, with the *k*_8_ fit value limited by the rate of dehydration. It is therefore possible that the cyclization rate of the B’ ring is greater than that of the A’ ring. *Thus, the main finding of these kinetic studies is that the faster cyclization of the A’ ring compared to dehydration of Ser18 precludes A-ring formation and is the key event that leads to a nonoverlapping ring pattern.*

### Dhb-Forming ProcA3.3 Variant Z Modification by ProcM

Variant Z has two types of changes compared to ProcA3.3. In order to independently determine the effect of the T11S and T18S mutations compared to the amino acid substitutions within the rings (A12F, G13N, K19F, and M20 V) on the final ring patterns, a ProcA3.3 variant Z analogue was generated that harbors S11T and S18T mutations (Figure 2D,E). This analogue leads to methyllanthionines similar to the WT ProcA3.3, and the changes compared to WT are just the substitutions within the ring. This substrate was utilized for in vitro reactions with ProcM under the same conditions as WT and variant Z, and the samples were subjected to LC-MS and tandem MS analysis as described in the Methods section. The total ion chromatographs (TIC) of the product peptide indicated two distinct peaks that correspond to overlapping and nonoverlapping ring patterns based on MS/MS fragmentation analysis (Figure S4). Several of the intermediate species were also subjected to MS/MS in an attempt to determine the location of the modifications. The analysis indicated a complex mixture of peptide modification sites. As a result, the MeLan-forming analogue of ProcA3.3 variant Z was not subjected to kinetic analysis, as it would be impossible to define a minimal kinetic model without knowing distinct modification sites. Similarly, we also generated another analogue of variant Z that retained the Ser residues at positions 11 and 18 but that had all other residues the same as WT ProcA3.3 (i.e., compared to WT, the only differences were Lan vs MeLan). Once again, this analogue resulted in a product mixture of overlapping and nonoverlapping rings (Figure S5) that was not further investigated. *Collectively, these results show that the change from overlapping to nonoverlapping ring patterns of the final products in going from WT to variant Z is caused by both the switch from MeLan to Lan rings and the substitution of residues in the rings, with the combined effect resulting in a complete switch to a nonoverlapping ring pattern.*

### Single Ring Cyclization Kinetics

The ring pattern of the product(s) formed by ProcM from ProcA3.3 and its variants is determined by the relative rates of formation of the A, A’, and B’ rings. If the A ring is formed faster than both the A’ and B’ rings, the overlapping ring pattern will be formed. Conversely, if either the A’ or the B’ ring is formed faster than the A ring, the nonoverlapping ring pattern will be generated. To determine if patterns for fully modified substrate peptides could be predicted by the relative rates of ring formation in isolation, peptides were designed to report on only one of the four possible rings per peptide (Figure S6). In these variants, all modifiable residues not involved in the formation of the ring of interest were mutated to alanine. We elected to do this only for the WT and variant Z ProcA3.3 peptides since they form products with single ring patterns. The variants were subjected to the same reaction conditions with ProcM, and the reactions were analyzed via LC-MS.

### Single Ring Cyclization Kinetics for ProcA3.3 WT

**Figure 5:**
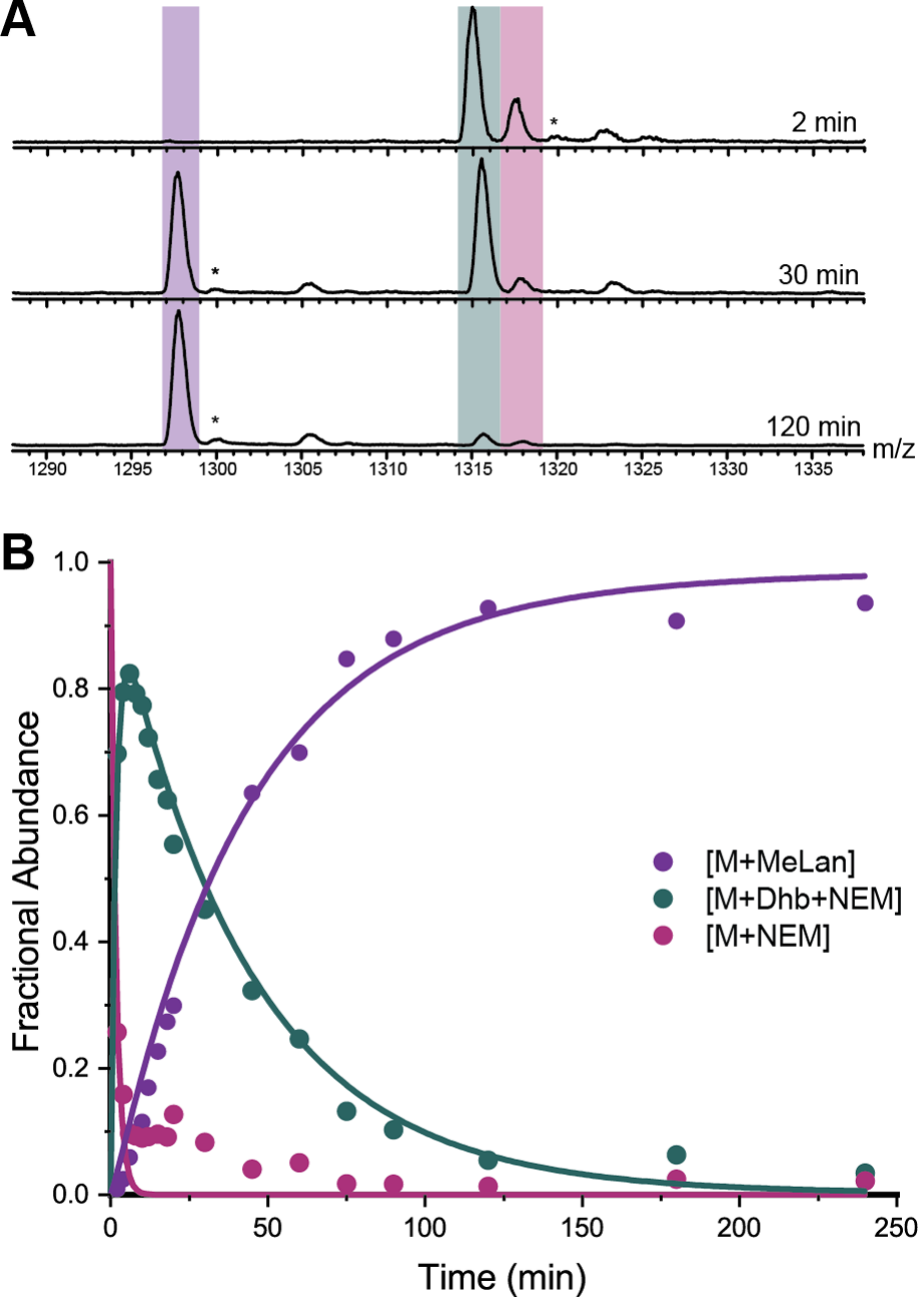
Representative example of kinetic data and fit for a ProcA3.3 variant that makes a single ring (B ring). Reactions for this figure were performed with ProcA3.3 B ring variant (ProcA3.3 T3A/C14A/T18A) at 40 μM substrate and 2 μM ProcM concentrations. (A) Representative ESI mass spectra of the 7+ charge state of the variant that forms the ProcA3.3 B ring at multiple time points. Coloring of each ion: dark magenta, unmodified peptide (M + NEM); teal, dehydrated peptide (M + 1 Dhb + NEM); purple, cyclized peptide (M + MeLan). (B) Time course for the intermediates and product of the ProcA3.3 B ring formation with fractional abundances for unmodified peptide (dark magenta), dehydrated peptide (teal), and cyclized peptide (purple). Peaks labeled with an asterisk (*) are oxidation products that were present in the starting peptide and that were carried through to oxidized products.

**Scheme 2:**
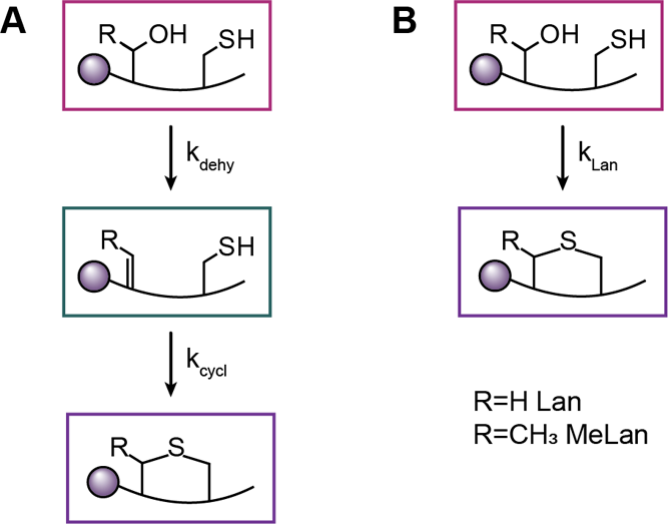
Proposed Minimal Kinetic Models for Isolated Ring Formation Both models contain unmodified peptide (dark magenta) and cyclized peptide (purple). (A) Minimal kinetic model in which dehydrated peptide intermediate (teal) is present. This model was used for the formation of A, B, and B’ rings for WT ProcA3.3 and the A, A’ ring of the ProcA3.3 variant Z. (B) Minimal kinetic model in which dehydrated intermediate was not present in sufficient quantity to use the former model with well-constrained values of rate constants. This model was used for the B’ ring of ProcA3.3 variant Z.

Single ring kinetic data were collected for variants of the WT ProcA3.3 scaffold that were only able to create the A, B, A’, and B’ rings (Figure S6). Peaks corresponding to unmodified, dehydrated, and cyclized peptides were monitored, and fractional abundance was again calculated to monitor the reaction progression (Figures 5 and S7–S9). These data were used to generate a minimal kinetic model in which the precursor peptide is modified to a MeLan ring-containing peptide through a dehydration step, *k*_dehy_, and a cyclization step, *k*_cycl_ (Scheme 2A). The use of previously determined binding constants was again applied to these models.

Analysis of the formation of individual rings in WT ProcA3.3 indicated that the rates for ring formation varied considerably (Table 2). The A ring, which is formed first in WT ProcA3.3, was formed the fastest in the single ring variants with a cyclization rate of 20 min^–1^. The B’ ring, which is the C terminal nonoverlapping ring, was the next fastest with a rate of 1.58 min^–1^, followed by the B ring which had the slowest measured rate of 0.53 min^–1^. These rate constants follow qualitative observations previously reported for WT ProcA3.3 that demonstrated that the A ring was formed first.^44^ Furthermore, when Cys14 involved in A ring formation was capped with a photolabile protecting group, the B’ ring was observed to form rather than the B ring.^45^ Thus, these individual ring cyclization rates are consistent with the experimentally observed ring cyclization order for WT ProcA3.3.

**Table 2:** Summary of the Simulated Rate Constants for Isolated Lanthipeptide Rings

isolated ring	*k*_*x*_	best fit ± SE (min^–1^)	FitSpace boundaries (min^–1^)^a^	*k*_net_ (μM^–1^ min^–1^)^b^
**ProcA3.3 WT**				
A ring (Dhb)	*k*_dehy_	9.1 ± 0.8	7.4–11.5	1.3
	*k*_cycl_	20 ± 4	14–37	2.1
B ring (Dhb)	*k*_dehy_	23 ± 4	15–50	2.3
	*k*_cycl_	0.53 ± 0.03	0.46–0.61	0.11
B’ ring (Dhb)	*k*_dehy_	11 ± 1	8–15	1.5
	*k*_cycl_	1.58 ± 0.09	1.33–1.86	0.312
A’ ring (Dhb)	*k*_dehy_	15.2 ± 0.7	12–21	1.84
	*k*_cycl_	0.024 ± 0.002	0.015–0.031	0.005
**ProcA3.3 variant Z**				
A ring (Dha)	*k*_dehy_	8.3 ± 0.5	7.4–8.9	1.2
	*k*_cycl_	32 ± 7	28–60	2.8
A’ ring (Dha)	*k*_dehy_	16 ± 2	14–18	1.9
	*k*_cycl_	120 ± 90	70–1090	3.7
B’ ring (Dha)	*k*_lan_	46 ± 4	39–58	3.0
A ring (Dhb)	*k*_dehy_	6.9 ± 0.5	6.1–8.1	1.1
	*k*_cycl_	11 ± 2	8–18	1.5

a FitSpace boundaries are reported for a χ^2^ threshold boundary of 0.83 for ProcA3.3 WT and 0.96 for the ProcA3.3 variant.

b *k*_net_= (*k*_on_*k*_*x*_)/(*k*_off_ + *k*_*x*_); *k*_on_ and *k*_off_ values were used from previously published data.^46^

Surprisingly, the isolated A’ ring was not efficiently cyclized by ProcM under these reaction conditions (Figure S10) even though this ring is formed in the presence of the B’ ring.^44^ This observation suggests that the presence of the B’ ring significantly accelerates the formation of the A’ ring within the WT ProcA3.3 sequence. *Collectively, the cyclization rates of individual rings correctly predict that WT ProcA3.3 would generate an overlapping ring pattern from the twice dehydrated intermediate (pink) in*Scheme 1A.

### Single Ring Cyclization Kinetics for ProcA3.3 Variant Z

Single cyclization kinetics were also performed on peptides only able to form the A, A’, and B’ rings in the ProcA3.3 variant Z scaffold (Figures S11–13). The B ring variant was not tested in isolation for this scaffold because this ring has never been observed to form first in any fully modified ProcA3.3 peptide. Peaks corresponding to unmodified, dehydrated, and cyclized peptide species were observed, and fractional abundance was calculated to monitor reaction progression. These data were used in the same minimal kinetic model as the ProcA3.3 WT isolated rings (Scheme 2A), except for the B’ ring of ProcA3.3 variant Z. For this substrate (like what was observed above for this ring in ProcA3.3 variant Z), the dehydrated intermediate was formed and consumed rapidly, resulting in a fit to the model in Scheme 2A that did not yield well-constrained rate constants. In order to generate better constrained rate constants, a second model was generated (Scheme 2B). In this model, the dehydrated intermediate was removed, and the dehydration and cyclization rates combined to give a lanthionine formation rate, *k*_lan_. In this model, *k*_dehy_ and *k*_cycl_ are coupled such that *k*_lan_ would be representative of the rate-limiting chemical modification, which is the dehydration rate.

Similar to the observations with the ProcA3.3 WT scaffold, the rate of isolated lanthionine formation varied widely (Table 2). The rings present in the fully modified peptide, A’ and B’, were formed at rates that showed minimal buildup of the dehydrated intermediate, resulting in an inability to measure *k*_cycl_ for the B’ ring as discussed above and a large standard error for the A’ ring cyclization rate. Examining the rate boundaries using the FitSpace Explorer module of KinTek indicates that the A’ ring *k*_cycl_ rate has a well-constrained lower boundary (70 min^–1^) and a poorly defined upper boundary (1090 min^–1^). Therefore, it is not possible to directly compare the cyclization rates of the two rings. Given that the dehydration rate constant for the A’ ring (16 min^–1^) is less than that of the lanthionine formation rate for the B’ ring (89 min^–1^) and that the dehydration step is rate limiting for both ring formations, it is possible that the cyclization step of the B’ ring in isolation is faster than that of the A’ ring in isolation. *More importantly, the isolated A’ and B’ rings are both formed at faster rates than the A ring in the variant Z scaffold. Therefore, the isolated ring formation rates correctly predict the final ring pattern for both the ProcA3.3 WT and variant Z.*

### Kinetics of Lanthionine and Methyllanthionine Formation in the Same Sequence Context

Although many studies on lanthipeptides have shown that the biosynthetic machinery can accept Ser-to-Thr and Thr-to-Ser mutations leading to interchange of Lan and MeLan rings,^53−56^ these studies have typically been conducted on systems that have one lanthipeptide synthetase and one substrate in their natural settings. For these systems, substrate and enzymatic machinery have coevolved under strong evolutionary pressure to make a bioactive product. Changing Lan to MeLan or vice versa are minor changes (addition or removal of a methyl group) that are tolerated by these highly evolved biosynthetic systems. On the other hand, as has been discussed previously,^46,49^ the system under investigation here in which one enzyme has at least 30 different substrates cannot have evolved to very efficiently make a single product (hence also the small amounts of off-pathway products observed in this study). Much evidence has been accumulated, including the current investigation that the substrate sequence determines the outcome of catalysis for these diversity-generating systems.^49^ The experiments presented in this work show that interconversion of Lan to MeLan and vice versa is not as straightforward for these systems and can lead to changes in the ring pattern formed. This finding raised a question that has not yet been addressed in lanthipeptide research: what is the difference in the rate of formation of Lan and MeLan within the same sequence context? We felt that the single ring kinetics presented above provided an excellent opportunity to investigate this question. Thus, we also performed kinetics for the formation of the MeLan A ring in the variant Z scaffold (Figure S14) to provide the first direct examination of the rate changes in enzyme-catalyzed cyclization of Lan versus MeLan. Similar to the other peptides, peaks corresponding to unmodified, dehydrated, and cyclized peptides were observed, and fractional abundance was calculated to monitor reaction progression (Figure S14). The data were used in the same minimal kinetic model as the other isolated ring formations with a dehydrated intermediate (Scheme 2A). The data show that the rates of dehydration of Ser or Thr in the same sequence context were near-identical (8.3 vs 6.9 min^–1^; Table 2), similar to the observations discussed earlier in this study. However, cyclization of the ring containing Dhb (11 min^–1^, Table 2) was about 3-fold slower than the Dha-containing counterpart (32 min^–1^), likely due to the increased steric hindrance and decreased electrophilicity.

## Conclusions

Kinetic analysis of ProcA3.3 WT and ProcA3.3 variant Z provided insights into why these two very similar peptides are converted into products with different ring patterns by the substrate-tolerant lanthipeptide synthetase ProcM. The data show that differences in the individual rates of the post-translational modifications (PTMs) resulted in changes in the order of the modifications, thus facilitating formation of certain rings that in turn prevent formation of other rings. In addition, the data in this work for ProcA3.3 suggest that several observations previously made for the modification of ProcA2.8 by ProcM may be general. First, the presence of prior PTMs alters the rate of modification within the peptide, with cyclizations generally slowing as more modifications are present. An exception is the A’ ring in WT ProcA3.3, the formation of which was considerably accelerated by the presence of the B’ ring. One possible explanation for these observations is that generally, the presence of a previously formed ring makes access to the active sites more difficult for subsequent modification, but for formation of the A’ ring in variant Z, the presence of the spatially distant nonoverlapping B’ ring is relatively innocuous and apparently even facilitates cyclization. The determination of the detailed kinetics of a second substrate, ProcA3.3, to complement the prior data on ProcA2.8 also shows that the kinetics of dehydration and cyclization for both substrates are quite similar. These observations support the previously proposed conformational selection model for diversity-generating lanthipeptide synthetases that convert a large number of sequence-diverse substrates into structurally diverse products.^46^ As shown previously with ProcA2.8 and now also for ProcA3.3, these systems differ from the lanthipeptide synthetases that have single substrates in that their catalytic activities are much slower, presumably because the enzymes did not coevolve with the single substrate to become highly specialized catalysts but instead retained high substrate tolerance for a large number of substrates but at the price of slower kinetics.^49^ Furthermore, including the data in the current study, ProcM has now been shown in several studies to form ring patterns that are dictated by the substrate sequence.^31^

Analysis of the rates of formation of isolated rings within the ProcA3.3 scaffold indicates that the relative individual ring formation rates correctly predict the final ring pattern, but the interplay of the relative rates of dehydration and cyclization reactions makes it difficult to predict outcomes without knowing the intrinsic rates of both dehydration and cyclization. The current studies on the ProcA3.3 scaffolds indicate that both the identities of the dehydratable residues and residues not directly involved in ring formation can be important for the ring pattern that will be formed. While the latter had been observed in previous studies,^31,49^ the notion that changing from Lan to MeLan (or vice versa) can change the ring pattern because of increased rates of formation of rings not present in the native product is an important unappreciated factor for predicting ring patterns, both for the products generated from novel peptide sequences in genomes and for library generation. The data in this study also illustrate the challenge of predicting ring patterns for uncharacterized peptide sequences encoded in the bacterial genomes since the relative rate constants for dehydration and cyclization are very context-dependent. High-throughput methods combined with machine learning may possibly provide sufficient data to achieve this goal.^57,58^

The findings in this work also provide important lessons regarding which substrates may be good for non-natural lanthipeptide library generation by avoiding product mixtures with different ring patterns. For ProcA3.3, the possibility to make the A-ring consisting of five amino acids or the A’/B’ rings with four amino acids, all of which are relatively fast processes, sets up a competition between formation of overlapping and nonoverlapping ring products. On the other hand, for ProcA2.8, which was successfully used for generation of a homogeneous library of products that all had the same ring pattern,^4^ the alternative to the native A and B ring, which are both seven-amino acid rings, would be to make two rings spanning 11 amino acids (Figure S19), which evidently was not a kinetically feasible possibility. These considerations also suggest that only a small subset of the 30 natural substrates for ProcM will be suitable for library generation. But with an estimated 1.6 million ProcA sequences encoded in the genomes and metagenomes as of 2017,^25^ additional suitable substrates can be identified when keeping these considerations in mind. The current study also provides a blueprint to investigate whether the conclusions reached here for ProcM are general for RiPP biosynthetic enzymes that generate polycyclic peptides such as radical-SAM enzymes and graspetide synthetases.^11^

## Supporting Information Available

Mass spectrometry data, kinetic data points and fits, confidence contours of the kinetic parameters, and observed and theoretical *m*/*z* values (PDF)

## References

[ref1] Newman D. J.; Cragg G. M. Natural products as sources of new drugs over the nearly four decades from 01/1981 to 09/2019. J. Nat. Prod. 2020, 83, 770–803. 10.1021/acs.jnatprod.9b01285. 32162523

[ref2] Ongpipattanakul C.; Desormeaux E. K.; DiCaprio A.; van der Donk W. A.; Mitchell D. A.; Nair S. K. Mechanism of action of ribosomally synthesized and post-translationally modified peptides. Chem. Rev. 2022, 122, 14722–14814. 10.1021/acs.chemrev.2c00210. 36049139 PMC9897510

[ref3] Urban J. H.; Moosmeier M. A.; Aumüller T.; Thein M.; Bosma T.; Rink R.; Groth K.; Zulley M.; Siegers K.; Tissot K.; et al. Phage display and selection of lanthipeptides on the carboxy-terminus of the gene-3 minor coat protein. Nat. Commun. 2017, 8, 1500 10.1038/s41467-017-01413-7. 29138389 PMC5686179

[ref4] Yang X.; Lennard K. R.; He C.; Walker M. C.; Ball A. T.; Doigneaux C.; Tavassoli A.; van der Donk W. A. A lanthipeptide library used to identify a protein-protein interaction inhibitor. Nat. Chem. Biol. 2018, 14, 375–380. 10.1038/s41589-018-0008-5. 29507389 PMC5866752

[ref5] Hetrick K. J.; Walker M. C.; van der Donk W. A. Development and application of yeast and phage display of diverse lanthipeptides. ACS Cent. Sci. 2018, 4, 458–467. 10.1021/acscentsci.7b00581. 29721528 PMC5920614

[ref6] Bosma T.; Rink R.; Moosmeier M. A.; Moll G. N. Genetically encoded libraries of constrained peptides. ChemBioChem. 2019, 20, 1754–1758. 10.1002/cbic.201900031. 30794341

[ref7] Vinogradov A. A.; Shimomura M.; Goto Y.; Ozaki T.; Asamizu S.; Sugai Y.; Suga H.; Onaka H. Minimal lactazole scaffold for in vitro thiopeptide bioengineering. Nat. Commun. 2020, 11, 2272 10.1038/s41467-020-16145-4. 32385237 PMC7210931

[ref8] King A. M.; Anderson D. A.; Glassey E.; Segall-Shapiro T. H.; Zhang Z.; Niquille D. L.; Embree A. C.; Pratt K.; Williams T. L.; Gordon D. B.; et al. Selection for constrained peptides that bind to a single target protein. Nat. Commun. 2021, 12, 6343 10.1038/s41467-021-26350-4. 34732700 PMC8566587

[ref9] Vinogradov A. A.; Zhang Y.; Hamada K.; Chang J. S.; Okada C.; Nishimura H.; Terasaka N.; Goto Y.; Ogata K.; Sengoku T.; et al. De Novo discovery of thiopeptide pseudo-natural products acting as potent and selective TNIK kinase inhibitors. J. Am. Chem. Soc. 2022, 144, 20332–20341. 10.1021/jacs.2c07937. 36282922 PMC9650704

[ref10] Repka L. M.; Chekan J. R.; Nair S. K.; van der Donk W. A. Mechanistic understanding of lanthipeptide biosynthetic enzymes. Chem. Rev. 2017, 117, 5457–5520. 10.1021/acs.chemrev.6b00591. 28135077 PMC5408752

[ref11] Montalbán-López M.; Scott T. A.; Ramesh S.; Rahman I. R.; van Heel A. J.; Viel J. H.; Bandarian V.; Dittmann E.; Genilloud O.; Goto Y.; et al. New developments in RiPP discovery, enzymology and engineering. Nat. Prod. Rep. 2021, 38, 130–239. 10.1039/D0NP00027B. 32935693 PMC7864896

[ref12] Eslami S. M.; van der Donk W. A. Proteases involved in leader peptide removal during RiPP biosynthesis. ACS Bio. Med. Chem. Au 2024, 4, 20–36. 10.1021/acsbiomedchemau.3c00059. PMC10885120 38404746

[ref13] Ortiz-López F. J.; Carretero-Molina D.; Sánchez-Hidalgo M.; Martín J.; González I.; Román-Hurtado F.; de la Cruz M.; García-Fernández S.; Reyes F.; Deisinger J. P.; et al. Cacaoidin, first member of the new lanthidin RiPP family. Angew. Chem., Int. Ed. 2020, 59, 12654–12658. 10.1002/anie.202005187. 32407589

[ref14] Xu M.; Zhang F.; Cheng Z.; Bashiri G.; Wang J.; Hong J.; Wang Y.; Xu L.; Chen X.; Huang S. X.; et al. Functional genome mining reveals a class V lanthipeptide containing a D-amino acid introduced by an F_420_H_2_-dependent reductase. Angew. Chem., Int. Ed. 2020, 59, 18029–18035. 10.1002/anie.202008035. 32648341

[ref15] Kloosterman A. M.; Cimermancic P.; Elsayed S. S.; Du C.; Hadjithomas M.; Donia M. S.; Fischbach M. A.; van Wezel G. P.; Medema M. H. Expansion of RiPP biosynthetic space through integration of pan-genomics and machine learning uncovers a novel class of lanthipeptides. PLoS Biol. 2020, 18, e3001026 10.1371/journal.pbio.3001026. 33351797 PMC7794033

[ref16] Hegemann J. D.; Süssmuth R. D. Matters of class: coming of age of class III and IV lanthipeptides. RSC Chem. Biol. 2020, 1, 110–127. 10.1039/D0CB00073F. 34458752 PMC8341899

[ref17] Román-Hurtado F.; Sánchez-Hidalgo M.; Martín J.; Ortiz-López F. J.; Genilloud O. Biosynthesis and heterologous expression of cacaoidin, the first member of the lanthidin family of RiPPs. Antibiotics 2021, 10, 403 10.3390/antibiotics10040403. 33917820 PMC8068269

[ref18] Liang H.; Lopez I. J.; Sánchez-Hidalgo M.; Genilloud O.; van der Donk W. A. Mechanistic studies on dehydration in class V lanthipeptides. ACS Chem. Biol. 2022, 17, 2519–2527. 10.1021/acschembio.2c00458. 36044589 PMC9486802

[ref19] Pei Z.-F.; Zhu L.; Sarksian R.; van der Donk W. A.; Nair S. K. Class V lanthipeptide cyclase directs the biosynthesis of a stapled peptide natural product. J. Am. Chem. Soc. 2022, 144, 17549–17557. 10.1021/jacs.2c06808. 36107785 PMC9621591

[ref20] Chatterjee C.; Miller L. M.; Leung Y. L.; Xie L.; Yi M.; Kelleher N. L.; van der Donk W. A. Lacticin 481 synthetase phosphorylates its substrate during lantibiotic production. J. Am. Chem. Soc. 2005, 127, 15332–15333. 10.1021/ja0543043. 16262372

[ref21] Dong S. H.; Tang W.; Lukk T.; Yu Y.; Nair S. K.; van der Donk W. A. The enterococcal cytolysin synthetase has an unanticipated lipid kinase fold. eLife 2015, 4, e07607 10.7554/eLife.07607. 26226635 PMC4550811

[ref22] Paul M.; Patton G. C.; van der Donk W. A. Mutants of the zinc ligands of lacticin 481 synthetase retain dehydration activity but have impaired cyclization activity. Biochemistry 2007, 46, 6268–6276. 10.1021/bi7000104. 17480057 PMC2517114

[ref23] Li B.; Sher D.; Kelly L.; Shi Y.; Huang K.; Knerr P. J.; Joewono I.; Rusch D.; Chisholm S. W.; van der Donk W. A. Catalytic promiscuity in the biosynthesis of cyclic peptide secondary metabolites in planktonic marine cyanobacteria. Proc. Natl. Acad. Sci. U.S.A. 2010, 107, 10430–10435. 10.1073/pnas.0913677107. 20479271 PMC2890784

[ref24] Zhang Q.; Yu Y.; Velásquez J. E.; van der Donk W. A. Evolution of lanthipeptide synthetases. Proc. Natl. Acad. Sci. U. S. A. 2012, 109, 18361–18366. 10.1073/pnas.1210393109. 23071302 PMC3494888

[ref25] Cubillos-Ruiz A.; Berta-Thompson J. W.; Becker J. W.; van der Donk W. A.; Chisholm S. W. Evolutionary radiation of lanthipeptides in marine cyanobacteria. Proc. Natl. Acad. Sci. U.S.A. 2017, 114, E5424–E5433. 10.1073/pnas.1700990114. 28630351 PMC5502607

[ref26] Arias-Orozco P.; Inklaar M.; Lanooij J.; Cebrián R.; Kuipers O. P. Functional expression and characterization of the highly promiscuous lanthipeptide synthetase SyncM, enabling the production of lanthipeptides with a broad range of ring topologies. ACS Synth. Biol. 2021, 10, 2579–2591. 10.1021/acssynbio.1c00224. 34554737 PMC8524650

[ref27] Arias-Orozco P.; Zhou L.; Yi Y.; Cebrián R.; Kuipers O. P.; Pidot S. J. Uncovering the diversity and distribution of biosynthetic gene clusters of prochlorosins and other putative RiPPs in marine *Synechococcus* strains. Microbiol. Spectr. 2024, 12, e0361123 10.1128/spectrum.03611-23. 38088546 PMC10783134

[ref28] Zhang Q.; Yang X.; Wang H.; van der Donk W. A. High divergence of the precursor peptides in combinatorial lanthipeptide biosynthesis. ACS Chem. Biol. 2014, 9, 2686–2694. 10.1021/cb500622c. 25244001 PMC4245175

[ref29] Burkhart B. J.; Kakkar N.; Hudson G. A.; van der Donk W. A.; Mitchell D. A. Chimeric leader peptides for the generation of non-natural hybrid RiPP products. ACS Cent. Sci. 2017, 3, 629–638. 10.1021/acscentsci.7b00141. 28691075 PMC5492250

[ref30] Hegemann J. D.; Bobeica S. C.; Walker M. C.; Bothwell I. R.; van der Donk W. A. Assessing the flexibility of the prochlorosin 2.8 scaffold for bioengineering applications. *ACS*. Synth. Biol. 2019, 8, 1204–1214. 10.1021/acssynbio.9b00080. PMC6525029 31042373

[ref31] Le T.; Jeanne Dit Fouque K.; Santos-Fernandez M.; Navo C. D.; Jiménez-Osés G.; Sarksian R.; Fernandez-Lima F. A.; van der Donk W. A. Substrate sequence controls regioselectivity of lanthionine formation by ProcM. J. Am. Chem. Soc. 2021, 143, 18733–18743. 10.1021/jacs.1c09370. 34724611 PMC8942616

[ref32] Wu C.; van der Donk W. A. Engineering of new-to-nature ribosomally synthesized and post-translationally modified peptide natural products. Curr. Opin. Biotechnol. 2021, 69, 221–231. 10.1016/j.copbio.2020.12.022. 33556835 PMC8238801

[ref33] Arias-Orozco P.; Yi Y.; Ruijne F.; Cebrián R.; Kuipers O. P. Investigating the specificity of the dehydration and cyclization reactions in engineered lanthipeptides by Synechococcal SyncM. ACS Synth. Biol. 2023, 12, 164–177. 10.1021/acssynbio.2c00455. 36520855 PMC9872173

[ref34] Do T.; Link A. J. Protein engineering in ribosomally synthesized and post-translationally modified peptides (RiPPs). Biochemistry 2023, 62, 201–209. 10.1021/acs.biochem.1c00714. 35006671 PMC9454058

[ref35] Eastman K. A. S.; Kincannon W. M.; Bandarian V. Leveraging substrate promiscuity of a radical *S*-adenosyl-L-methionine RiPP maturase toward intramolecular peptide cross-linking applications. ACS Cent. Sci. 2022, 8, 1209–1217. 10.1021/acscentsci.2c00501. 36032765 PMC9413430

[ref36] Si Y.; Kretsch A. M.; Daigh L. M.; Burk M. J.; Mitchell D. A. Cell-free biosynthesis to evaluate lasso peptide formation and enzyme-substrate tolerance. J. Am. Chem. Soc. 2021, 143, 5917–5927. 10.1021/jacs.1c01452. 33823110 PMC8062304

[ref37] Vinogradov A. A.; Yin Y.; Suga H. Macrocyclic peptides as drug candidates: recent progress and remaining challenges. J. Am. Chem. Soc. 2019, 141, 4167–4181. 10.1021/jacs.8b13178. 30768253

[ref38] Muttenthaler M.; King G. F.; Adams D. J.; Alewood P. F. Trends in peptide drug discovery. Nat. Rev. Drug Discovery 2021, 20, 309–325. 10.1038/s41573-020-00135-8. 33536635

[ref39] Tucker T. J.; Embrey M. W.; Alleyne C.; Amin R. P.; Bass A.; Bhatt B.; Bianchi E.; Branca D.; Bueters T.; Buist N.; et al. A series of novel, highly potent, and orally bioavailable next-generation tricyclic peptide PCSK9 inhibitors. J. Med. Chem. 2021, 64, 16770–16800. 10.1021/acs.jmedchem.1c01599. 34704436

[ref40] Philippe G. J. B.; Craik D. J.; Henriques S. T. Converting peptides into drugs targeting intracellular protein-protein interactions. Drug Discovery Today 2021, 26, 1521–1531. 10.1016/j.drudis.2021.01.022. 33524603

[ref41] Ji X.; Nielsen A. L.; Heinis C. Cyclic peptides for drug development. Angew. Chem., Int. Ed. 2024, 63, e202308251 10.1002/anie.202308251. 37870189

[ref42] Tang W.; van der Donk W. A. Structural characterization of four prochlorosins: a novel class of lantipeptides produced by planktonic marine cyanobacteria. Biochemistry 2012, 51, 4271–4279. 10.1021/bi300255s. 22574919 PMC3361976

[ref43] Bobeica S. C.; Zhu L.; Acedo J. Z.; Tang W.; van der Donk W. A. Structural determinants of macrocyclization in substrate-controlled lanthipeptide biosynthetic pathways. Chem. Sci. 2020, 11, 12854–12870. 10.1039/D0SC01651A. 34094481 PMC8163290

[ref44] Mukherjee S.; van der Donk W. A. Mechanistic studies on the substrate-tolerant lanthipeptide synthetase ProcM. J. Am. Chem. Soc. 2014, 136, 10450–10459. 10.1021/ja504692v. 24972336 PMC4111213

[ref45] Yu Y.; Mukherjee S.; van der Donk W. A. Product formation by the promiscuous lanthipeptide synthetase ProcM is under kinetic control. J. Am. Chem. Soc. 2015, 137, 5140–5148. 10.1021/jacs.5b01409. 25803126 PMC4487812

[ref46] Thibodeaux C. J.; Ha T.; van der Donk W. A. A price to pay for relaxed substrate specificity: a comparative kinetic analysis of the class II lanthipeptide synthetases ProcM and HalM2. J. Am. Chem. Soc. 2014, 136, 17513–17529. 10.1021/ja5089452. 25409537 PMC4277782

[ref47] Le T.; Zhang D.; Martini R. M.; Biswas S.; van der Donk W. A. Use of a head-to-tail peptide cyclase to prepare hybrid RiPPs. Chem. Commun. 2024, 60, 6508–6511. 10.1039/D3CC04919A. PMC11189026 38833296

[ref48] Mi X.; Desormeaux E. K.; Le T. T.; van der Donk W. A.; Shukla D. Sequence controlled secondary structure is important for the site-selectivity of lanthipeptide cyclization. Chem. Sci. 2023, 14, 6904–6914. 10.1039/D2SC06546K. 37389248 PMC10306099

[ref49] Le T.; van der Donk W. A. Mechanisms and evolution of diversity-generating RiPP biosynthesis. Trends Chem. 2021, 3, 266–278. 10.1016/j.trechm.2021.01.003.

[ref50] Meyrat A.; von Ballmoos C. ATP synthesis at physiological nucleotide concentrations. Sci. Rep. 2019, 9, 3070 10.1038/s41598-019-38564-0. 30816129 PMC6395684

[ref51] Johnson K. A.; Simpson Z. B.; Blom T. Global Kinetic Explorer: A new computer program for dynamic simulation and fitting of kinetic data. Anal. Biochem. 2009, 387, 20–29. 10.1016/j.ab.2008.12.024. 19154726

[ref52] Johnson K. A.; Simpson Z. B.; Blom T. FitSpace Explorer: An algorithm to evaluate multi-dimensional parameter space in fitting kinetic data. Anal. Biochem. 2009, 387, 30–41. 10.1016/j.ab.2008.12.025. 19168024

[ref53] Kuipers O. P.; Bierbaum G.; Ottenwälder B.; Dodd H. M.; Horn N.; Metzger J.; Kupke T.; Gnau V.; Bongers R.; van den Bogaard P.; et al. Protein engineering of lantibiotics. Antonie van Leeuwenhoek 1996, 69, 161–169. 10.1007/BF00399421. 8775976

[ref54] Ross A. C.; Vederas J. C. Fundamental functionality: recent developments in understanding the structure-activity relationships of lantibiotic peptides. J. Antibiot. 2011, 64, 27–34. 10.1038/ja.2010.136. 21081951

[ref55] Cotter P. D.; Hill C.; Ross R. P. Bacterial lantibiotics: strategies to improve therapeutic potential. Curr. Protein Pept. Sci. 2005, 6, 61–75. 10.2174/1389203053027584. 15638769

[ref56] Sarksian R.; van der Donk W. A. Divergent evolution of lanthipeptide stereochemistry. ACS Chem. Biol. 2022, 17, 2551–2558. 10.1021/acschembio.2c00492. 36001880 PMC9486935

[ref57] Vinogradov A. A.; Chang J. S.; Onaka H.; Goto Y.; Suga H. Accurate models of substrate preferences of post-translational modification enzymes from a combination of mRNA display and deep learning. ACS Cent. Sci. 2022, 8, 814–824. 10.1021/acscentsci.2c00223. 35756369 PMC9228559

[ref58] Chang J. S.; Vinogradov A. A.; Zhang Y.; Goto Y.; Suga H. Deep learning-driven library design for the de novo discovery of bioactive thiopeptides. ACS Cent. Sci. 2023, 9, 2150–2160. 10.1021/acscentsci.3c00957. 38033794 PMC10683472

