# Supplementary material for: Kinetic Analysis of Cyclization by the Substrate-Tolerant Lanthipeptide Synthetase ProcM
Source: ACS Catal. 2024 Nov 27;14(24):18310–21. doi: 10.1021/acscatal.4c06216 (PMC11667668; doi:10.1021/acscatal.4c06216)
Supplement: Supplementary file 1 — cs4c06216_si_001.pdf [file cs4c06216_si_001.pdf]

Supporting Information

Kinetic Analysis of Cyclization by the Substrate-Tolerant Lanthipeptide Synthetase ProcM

Emily K. Desormeaux[1], Garrett J. Barksdale[2], and Wilfred A. van der Donk[1,2,3,*]

[1]Department of Chemistry, [2]School of Molecular and Cellular Biology, and [3]Howard Hughes Medical Institute, University of Illinois at Urbana-Champaign, 600 South Mathews Avenue, Urbana, Illinois 61801, USA.

[*] vddonk@illinois.edu

Table of Contents

Figure S1. ProcA3.3 WT kinetics 4

Figure S2. Fragmentation analysis of singly cyclized intermediate for ProcA3.3 variant Z.......... 5

Figure S3. ProcA3.3 Variant Z kinetics........ 6

Figure S4. MeLan-forming ProcA3.3 Variant Z analysis 8

Figure S5. Dha containing ProcA3.3 WT analysis 9

Figure S6. ProcA3.3 peptides used for isolated ring kinetics 10

Figure S7. ProcA3.3 A ring kinetics........11

Figure S8. ProcA3.3 B ring kinetics........12

Figure S9. ProcA3.3 B’ ring kinetics 13

Figure S10. ProcA3.3 A’ ring kinetics 14

Figure S11. ProcA3.3 Variant Z A ring kinetics........15

Figure S12. ProcA3.3 Variant Z A’ ring kinetics........16

Figure S13. ProcA3.3 Variant Z B’ ring kinetics........17

Figure S14. ProcA3.3 Variant Z MeLan A ring kinetics........18

Figure S15. MALDI-ToF mass spectra for parent ProcA3.3 peptides. 19

Figure S16. MALDI-ToF mass spectra for peptides used for the ProcA3.3 WT scaffold isolated ring analysis.........20

Figure S17. MALDI-ToF mass spectra for peptides used for the ProcA3.3 variant Z scaffold isolated ring analysis.........21

Figure S18. MALDI-ToF mass spectra for peptides used for Lan vs MeLan analysis. 22

Figure S19. Ring patterns of various characterized prochlorosins. 23

Table S1. Peak assignments for the peptide ions observed in thrombin digested substrates for the indicated ProcM-catalyzed reaction.........24

References 24

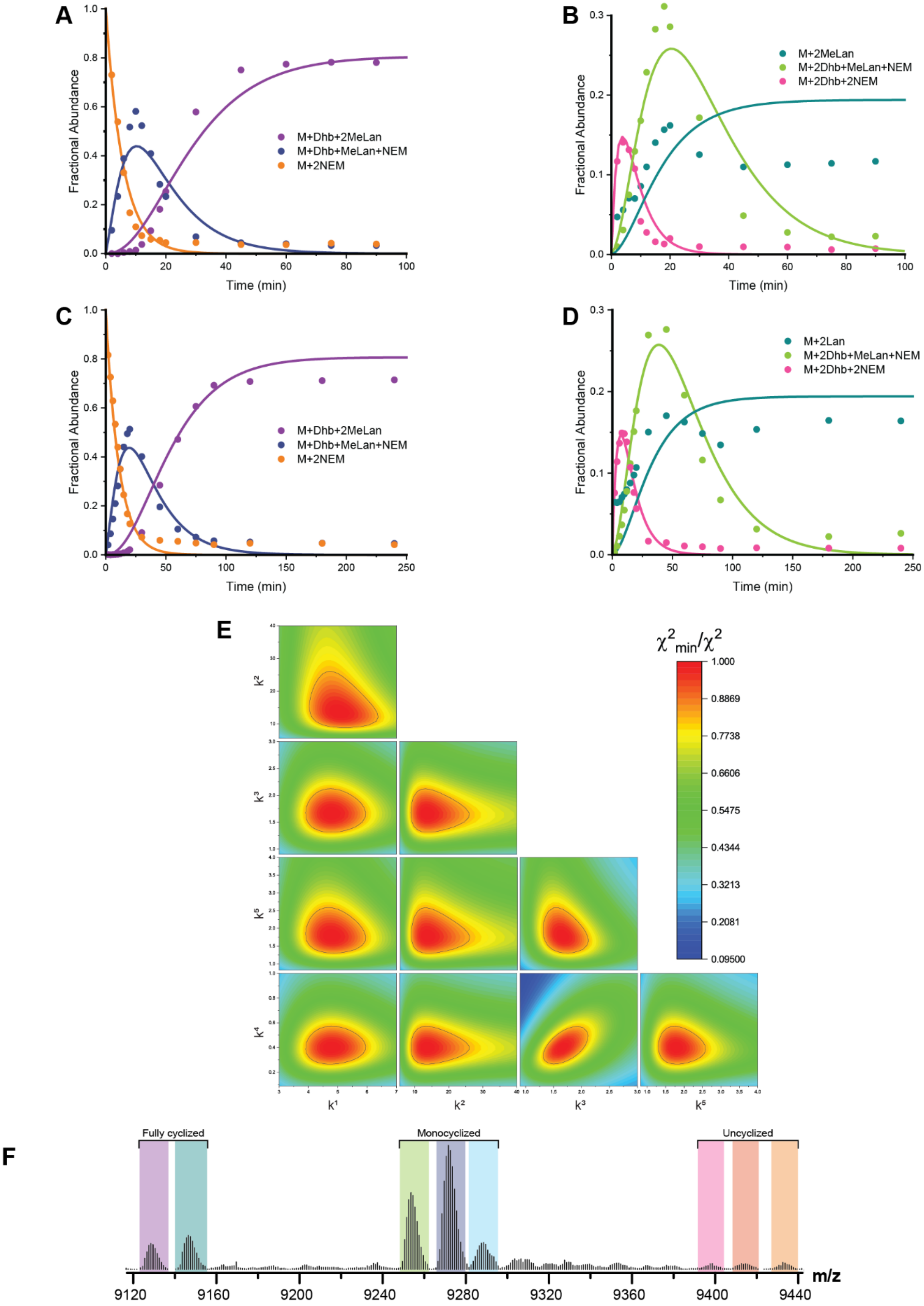
A
Fractional Abundance
Time (min)
M+Dhb+2MeLan
M+Dhb+MeLan+NEM
M+2NEM
B
M+2MeLan
M+2Dhb+MeLan+NEM
M+2Dhb+2NEM
C
D
M+2Lan
E
χ^2_{min}/χ^2
1.000
0.8869
0.7738
0.6606
0.5475
0.4344
0.3213
0.2081
0.09500
k¹
k²
k³
k⁵
k⁴
F
Fully cyclized
Monocyclized
Uncyclized
m/z
9120
9160
9200
9240
9280
9320
9360
9400
9440

G

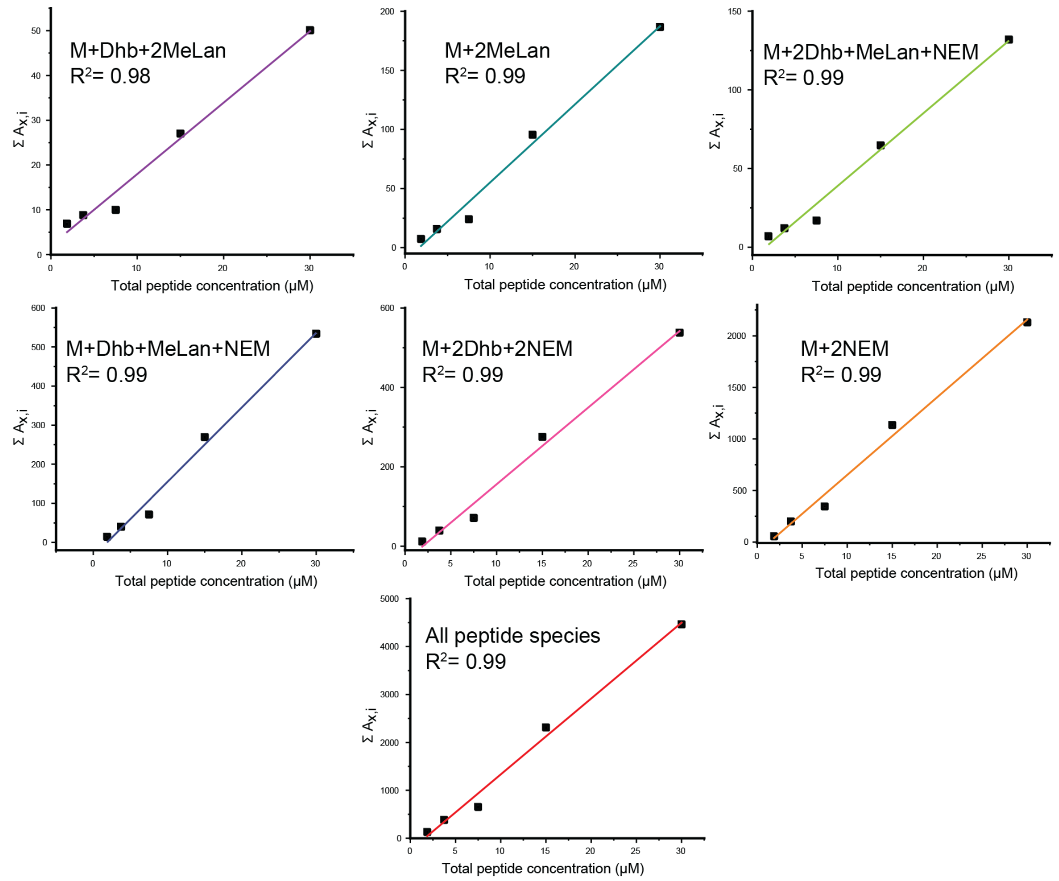

H

`GSHMSEEQLKAFIAKVQGDSSLQEQLKAEGADVVAIAKAAGFTIKQQDLNAAASELSDEELEAASGG-Core peptide`

Figure S1. ProcA3.3 WT kinetics. Species observed include unmodified peptide (orange), peptide containing one MeLan ring and one dehydration (dark blue), the final product (purple), uncyclized peptide with two dehydrations (pink), peptide containing one MeLan ring with two dehydrations (green), and peptide containing two MeLan rings with no dehydrations (cyan). **A/B)** Time course for species in a reaction containing 40 μM peptide and 2 μM ProcM. **C/D)** Time course for species for reaction containing 80 μM peptide and 2 μM ProcM. **E)** Confidence contours used to evaluate the upper and lower bounds for each rate constant listed in Table 1 of the main text. The kinetic data shown here and in Figure 3 of the main text were simulated using the kinetic model shown in Scheme 1A. The fit of the rate constants listed in Table 1 yielded a χ_{min}^2 of 131.8 (χ^2/DOF = 0.438). The confidence contours indicate the χ_{min}^2/χ^2 value as a function of variable parameters indicated on the x and y axis. Values with a χ_{min}^2/χ^2 value greater than 0.833 are within the black line indicated on the plot. This is the cut off value that was utilized for all ProcA3.3 WT reactions. **F)** Representative deconvoluted ESI mass spectra of ProcA3.3 WT reaction with ProcM at 20 min of the 60 μM reaction. Data has been represented in centroid mode to show monoisotopic species as opposed to profile mode as shown in Figure 3A. See Table S1 for a full listing of calculated and observed m/z values for all ions. For each trace, the y-axis was scaled to the intensity of the highest peak present. The sample was treated with NEM to interrogate the cyclization state of the Cys residues. The coloring of each ion matches those in other panels and those in Figure 3. **G)** Linear fits of LCMS EIC peak area vs total peptide concentration for ProcA3.3 derived peptides. **H)** Sequence of the leader peptide after thrombin digestion of His_6-ProcA3.3. This peptide is present in all LCMS assays performed and explains why the ionization efficiencies of peptides were very similar.

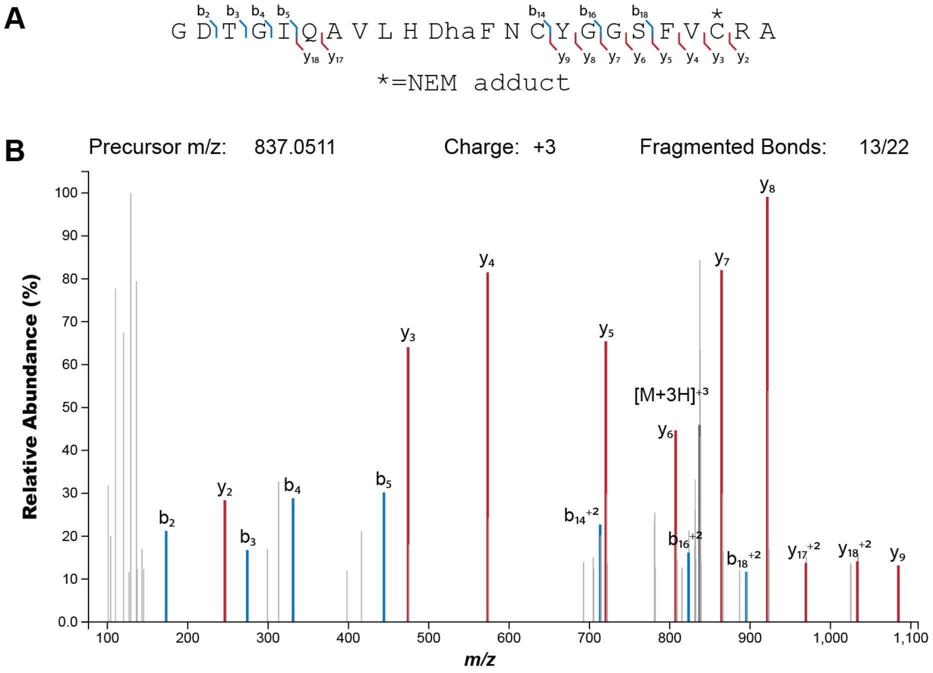

C

Ion	Calc. mass	Obs. Mass	Error (ppm)
b_2^+	173.0557	173.0557	0.1
y_2^+	246.1563	246.1561	1.0
b_3^+	274.1054	274.1034	7.4
b_4^+	331.1248	331.1248	-0.1
b_5^+	444.2091	444.2089	0.5
y_3^+	474.2121	474.213	-1.8
y_4^+	573.2797	573.2814	-2.9
b_{14}^{+2}	713.3316	713.3326	-1.4
y_5^+	720.3483	720.3498	-2.1
y_6^+	807.3811	807.3818	-0.9
b_{16}^{+2}	823.3737	823.375	-1.6
$[M+3H]^{+3}$	837.0498	837.0511	-1.6
y_7^+	864.4013	864.4033	-2.3
b_{18}^{+2}	895.3987	895.4018	-3.4
y_8^+	921.4233	921.4247	-1.6
y_{17}^{+2}	969.4404	969.4429	-2.6
y_{18}^{+2}	1033.4737	1033.4722	1.4
y_9^+	1084.4844	1084.4881	-3.4

Figure S2. Fragmentation analysis of singly cyclized intermediate for ProcA3.3 variant Z. **A)** Sequence of ProcA3.3 variant singly cyclized intermediate after leader peptide cleavage with $LahT_{150}$.[1] Observed fragmentation is indicated with labeled y and b ions. **B)** Tandem-MS fragmentation of spectrum with corresponding peaks labeled. **C)** Plot of observed errors for each peak calculated in ppm. Panels A and B are modified from those produced on Interactive Peptide Spectral Annotator.[2]

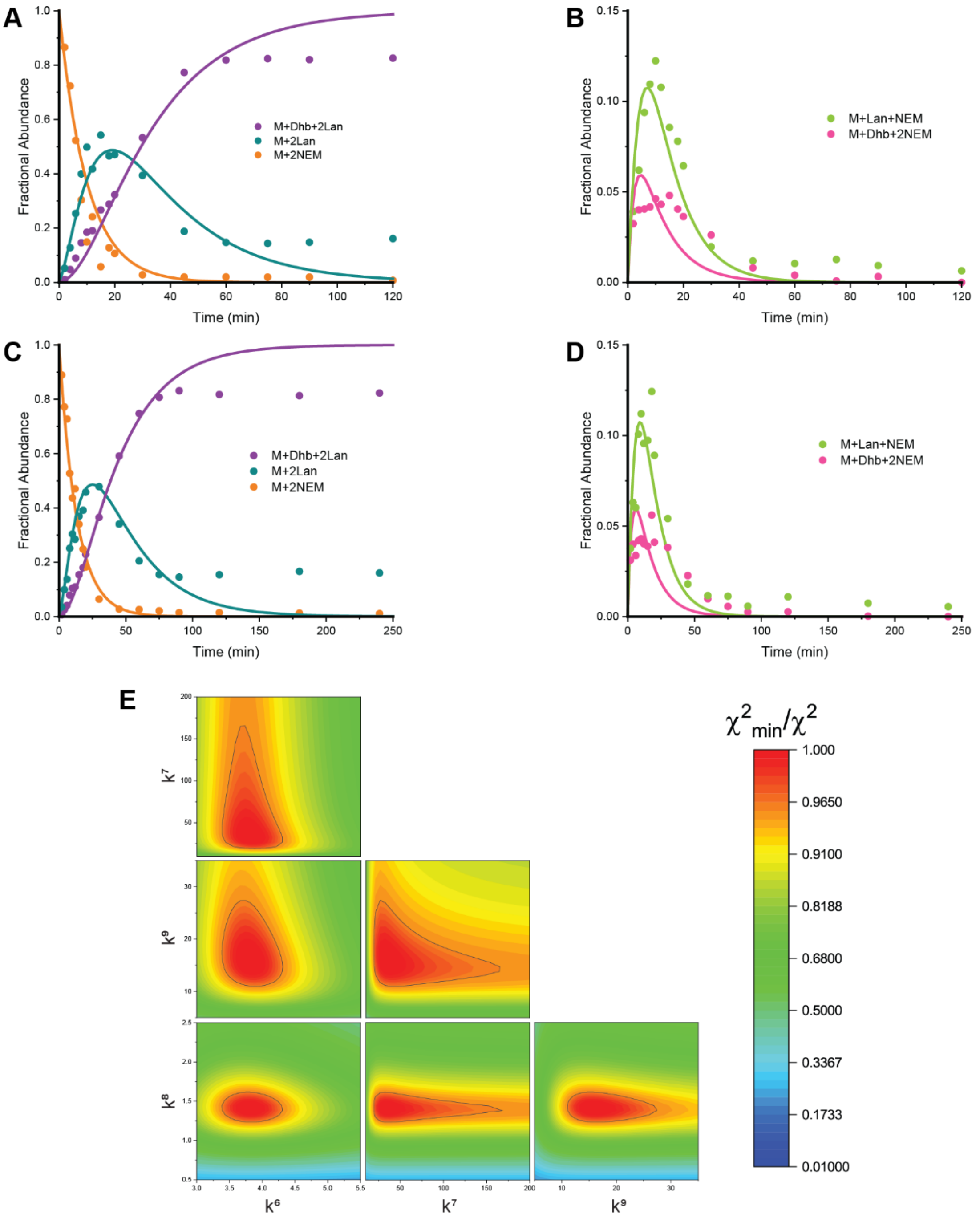

Figure S3. ProcA3.3 Variant Z kinetics. Species observed include unmodified peptide (orange), peptide containing two Lan rings with no additional dehydrations (cyan), the final product (purple), uncyclized peptide with one dehydration (pink), and peptide containing one Lan ring (green). **A/B)** Time course for species for the reaction containing 60 μM peptide and 2 μM ProcM. **C/D)** Time course for the reaction containing 80 μM peptide and 2 μM ProcM. **E)** Confidence contours used to evaluate the upper and lower bounds for each rate constant listed in Table 1 of the main text.

The kinetic data shown here and in Figure 4 of the main text were simulated using the kinetic model shown in Scheme 1B. The fit of the rate constants listed in Table 1 yielded a χ_{min}^2 of 394.7 (χ^2/DOF = 1.573). The confidence contours indicate the $\chi_{min}^2/\ \chi^2$ value as a function of variable parameters indicated on the x and y axis. Values with a $\chi_{min}^2/\ \chi^2$ value greater than 0.96 are within the black line indicated on the plot. This is the cut off value that was utilized for all ProcA3.3 variants. All rate constants within Scheme 1B were well-defined by the data collected, however the k_7 rate constants have well defined lower boundaries, with less well-defined upper boundaries.

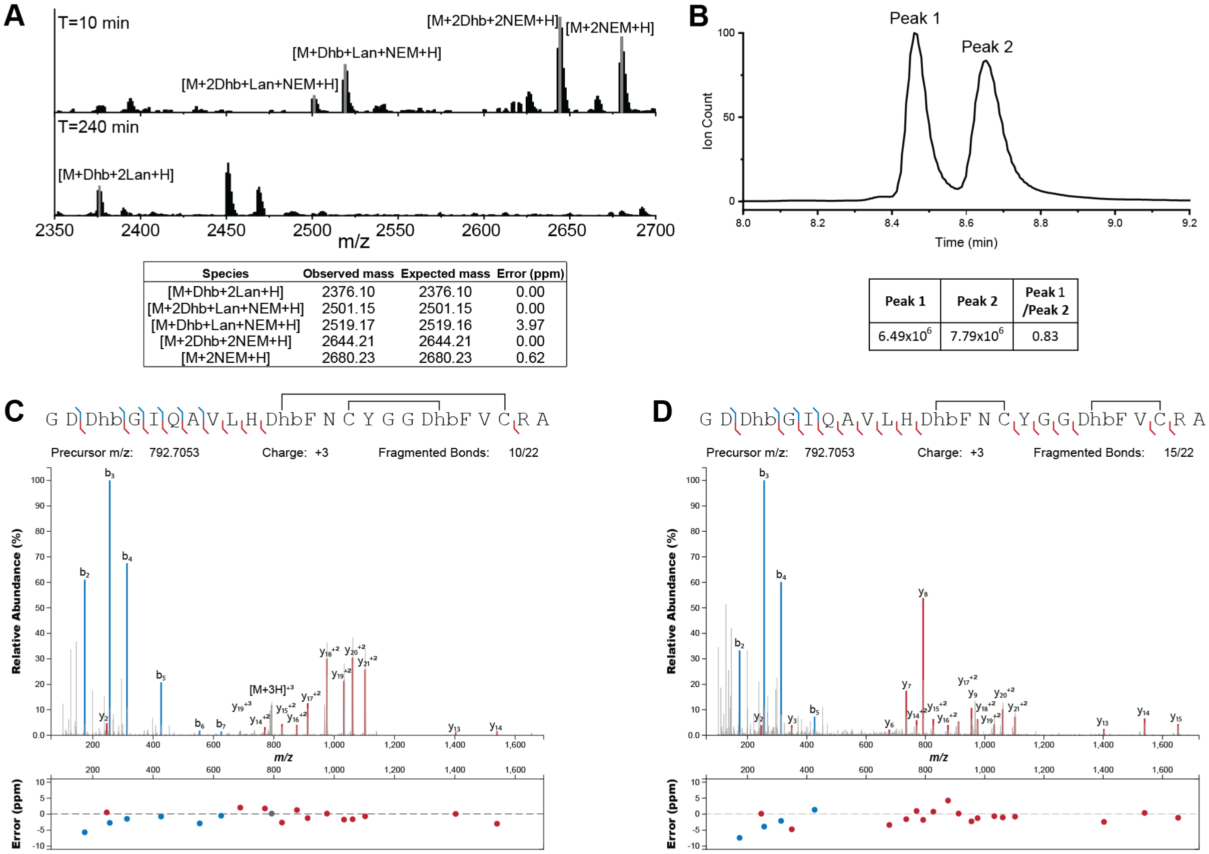

Species	Observed mass	Expected mass	Error (ppm)
[M+Dhb+2Lan+H]	2376.10	2376.10	0.00
[M+2Dhb+Lan+NEM+H]	2501.15	2501.15	0.00
[M+Dhb+Lan+NEM+H]	2519.17	2519.16	3.97
[M+2Dhb+2NEM+H]	2644.21	2644.21	0.00
[M+2NEM+H]	2680.23	2680.23	0.62

Peak 1	Peak 2	Peak 1 /Peak 2
6.49×10^6	7.79×10^6	0.83

Figure S4. MeLan-forming ProcA3.3 Variant Z analysis. A) Deconvoluted ESI mass spectra of MeLan-forming ProcA3.3 variant Z modified by ProcM at the 10- and 240-minute time points. The sample was treated with NEM to interrogate the cyclization state of the Cys residues. Peaks corresponding to peptide intermediates are labeled, and observed and expected masses are provided below the spectrum. **B)** Extracted ion chromatogram of MeLan containing ProcA3.3 variant Z products (m/z=792.7054, z=3) after an overnight reaction. **C/D)** Observed fragmentation is indicated with labeled y and b ions of both peptides associated with peaks 1 and 2 of the extracted ion chromatogram (panel B), respectively. Error is depicted below for each corresponding labeled peak above. Panels C and D were produced using Interactive Peptide Spectral Annotator.[2] The fragmentation observed indicates that peak 1 corresponds to product with an overlapping ring pattern and peak 2 corresponds to product with a non-overlapping ring pattern. Samples for all panels were treated with $LahT_{150}$,[1] and protein was precipitated via MeOH addition prior to analysis (see methods section).

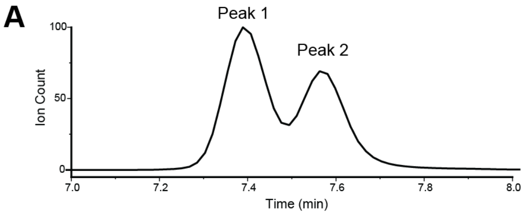

Peak 1	Peak 2	Peak 1 /Peak 2
8.09x10⁵	6.02x10⁵	1.34

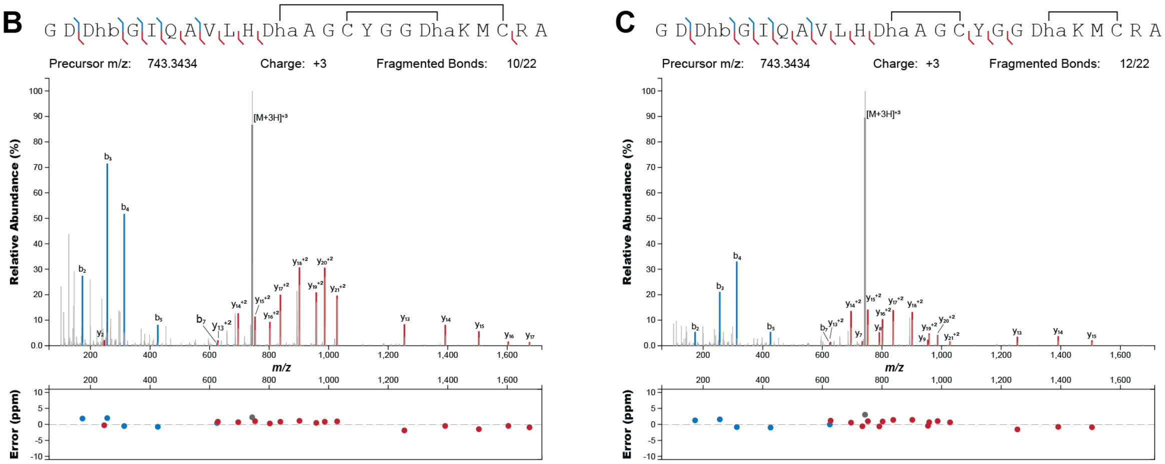

Figure S5. Lan-forming ProcA3.3 WT analysis. A) Extracted ion chromatogram of products formed from Lan-forming ProcA3.3 WT (m/z=743.3435, z=3) after the conclusion of a four-hour reaction. **B/C)** Observed fragmentation is indicated with labeled y and b ions of both Peak 1 and 2 of the extracted ion chromatogram, respectively. Error is depicted below for each corresponding labeled peak above. Panels B and C were produced using Interactive Peptide Spectral Annotator.[2] Fragmentation indicates that peak 1 corresponds to the overlapping ring pattern and peak 2 corresponds to the non-overlapping ring pattern. Samples for all panels were treated with $LahT_{150}$, and protein was precipitated via MeOH addition prior to analysis (see methods section).

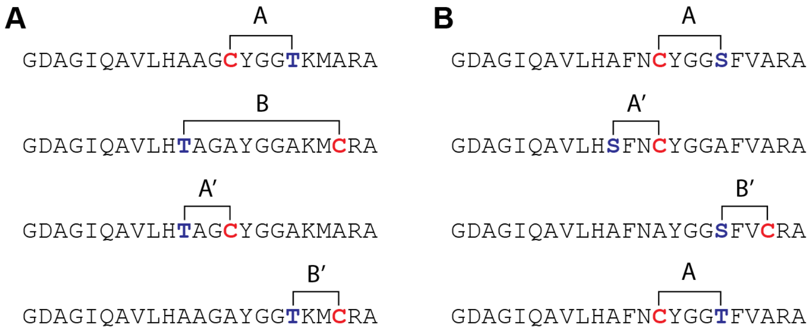

Figure S6. ProcA3.3 peptides used for isolated ring kinetics. **A)** The four variants used for kinetic analysis of individual cyclization rates on the ProcA3.3 WT scaffold. **B)** The four variants used for kinetic analysis of individual cyclization rates on the ProcA3.3 variant Z scaffold. A peptide that would form the B ring was not utilized for kinetic studies for this variant scaffold since this ring is never formed first.

We note that in making single ring variants we introduced Ala mutations at the Ser/Thr/Cys residues that are not involved in the ring of interest. Those mutations could affect the rate constants. For most steps that occur in the corresponding parent substrates that make multiple rings, we cannot determine whether these mutations affected the rates because the mutations prevent these steps. We can compare the kinetics of a few steps. For instance, dehydration of Thr18 in the A-ring substrate and in the B'-ring substrates (WT ProcA3.3 scaffold) are 11 and 9.1 min^{-1} respectively, suggesting that the replacement of either Cys21 or Cys14 with Ala, respectively, essentially had no influence on the rate of dehydration (see Figures S7 and S9). Furthermore, cyclization of the A-ring in WT ProcA3.3 (which is the first ring formed; 14 min^{-1}) is similar to that of the single A-ring substrate (20 min^{-1}). However, we do see some examples where these mutations do make difference. In the variant Z scaffold, dehydration of Ser18 for the single A-ring substrate (8.3 min^{-1}, Fig. S11) is considerably slower than the dehydration of Ser18 for the single B'-ring substrate (46 ± 4 min^{-1}, Fig. S13). These are inherent limitations of the approach.

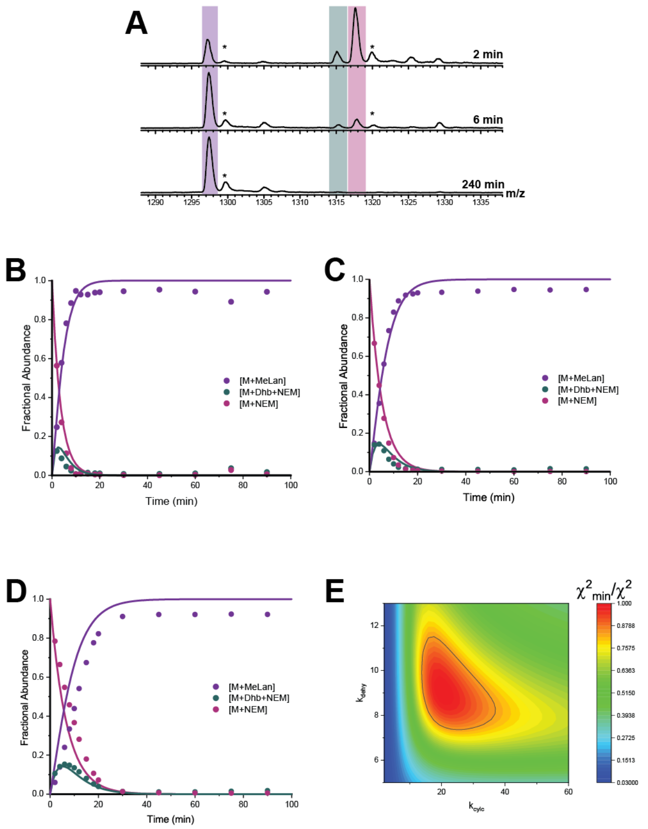

Figure S7. ProcA3.3 A ring kinetics. A) Representative ESI mass spectra of the 8+ charge state at multiple time points. For each trace, the y-axis is scaled to the intensity of the highest peak present. The sample was treated with NEM to interrogate the cyclization state of the Cys residue. Species observed include unmodified (pink), dehydrated (teal), and cyclized (purple) peptide. **B)** Time course for reaction containing 40 µM peptide and 2 µM ProcM. **C)** Time course for reaction containing 60 µM peptide and 2 µM ProcM. **D)** Time course for reaction containing 80 µM peptide and 2 µM ProcM. **E)** Confidence contour for the cyclization of the ProcA3.3 A ring in isolation was calculated with rate constants listed in Table 2 of main text. The kinetic data shown here were simulated using the kinetic model shown in Scheme 2A of the main text. The fit of the rate constants listed in Table 2 yielded a χ_{min}^2 of 1813.9 (χ^2/DOF = 12.3). The confidence contours indicate the χ_{min}^2/ χ^2 value as a function of variable parameters indicated on the x and y axis. Values with a χ_{min}^2/ χ^2 value greater than 0.833 are within the black line indicated on the plot. Peaks labeled with an asterisk (*) are oxidation products that were present in the starting peptide and that were carried through to oxidized products.

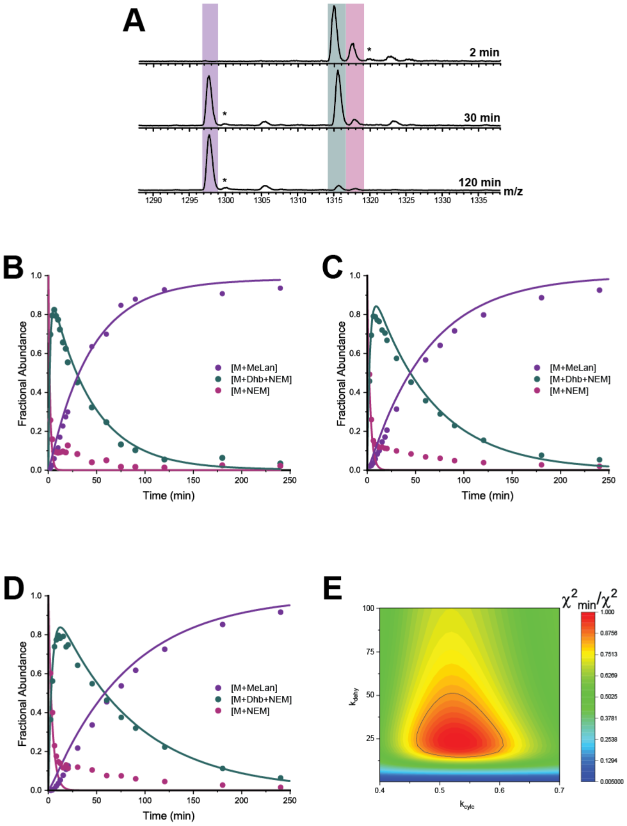

Figure S8. ProcA3.3 B ring kinetics. A) Representative ESI mass spectra of the 8+ charge state at multiple time points. For each trace, the y-axis is scaled to the intensity of the highest peak present. The sample was treated with NEM to interrogate the cyclization state of the Cys residue. Species observed include unmodified (pink), dehydrated (teal), and cyclized (purple) peptide. **B)** Time course for reaction containing 40 µM peptide and 2 µM ProcM. **C)** Time course for reaction containing 60 µM peptide and 2 µM ProcM. **D)** Time course for reaction containing 80 µM peptide and 2 µM ProcM. **E)** Confidence contour for the cyclization of the ProcA3.3 B ring in isolation was calculated with rate constants listed in Table 2 of main text. The kinetic data shown here were simulated using the kinetic model shown in Scheme 2A of the main text. The fit of the rate constants listed in Table 2 yielded a χ_{min}^2 of 800.9 (χ^2/DOF = 5.3). The confidence contours indicate the χ_{min}^2/χ^2 value as a function of variable parameters indicated on the x and y axis. Values with a χ_{min}^2/χ^2 value greater than 0.833 are within the black line indicated on the plot. Peaks labeled with an asterisk (*) are oxidation products that were present in the starting peptide and that were carried through to oxidized products.

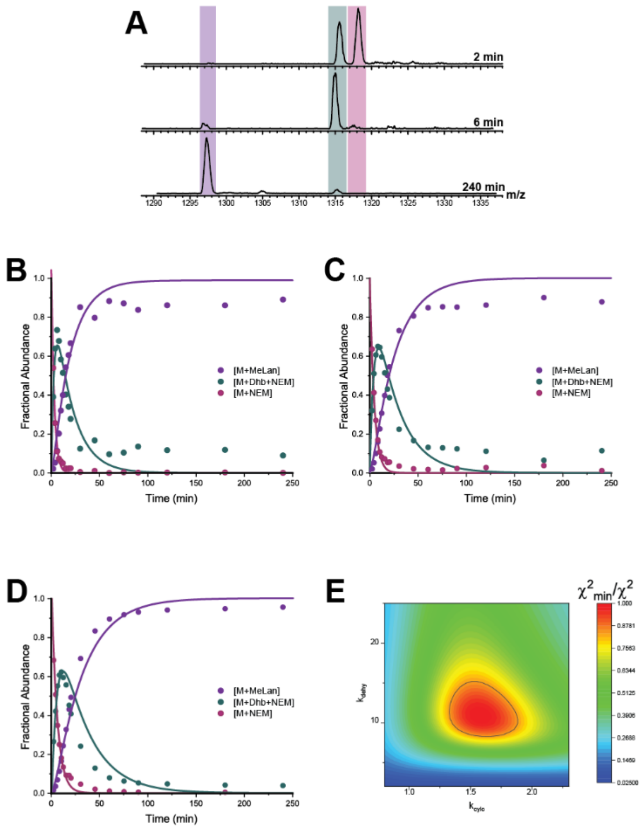

Figure S9. ProcA3.3 B' ring kinetics. A) Representative ESI mass spectra of the 8+ charge state at multiple time points. For each trace, the y-axis is scaled to the intensity of the highest peak present. The sample was treated with NEM to interrogate the cyclization state of the Cys residue. Species observed include unmodified (pink), dehydrated (teal), and cyclized (purple) peptide. **B)** Time course for reaction containing 40 μM peptide and 2 μM ProcM. **C)** Time course for reaction containing 60 μM peptide and 2 μM ProcM. **D)** Time course for reaction containing 80 μM peptide and 2 μM ProcM. **E)** Confidence contour for the cyclization of the ProcA3.3 B' ring in isolation was calculated with rate constants listed in Table 2 of main text. The fit of the rate constants listed in Table 2 yielded a χ_{min}^2 of 396 (χ^2/DOF = 2.6). The kinetic data shown here were simulated using the kinetic model shown in Scheme 2A of the main text. The confidence contours indicate the χ_{min}^2/ χ^2 value as a function of variable parameters indicated on the x and y axis. Values with a χ_{min}^2/ χ^2 value greater than 0.833 are within the black line indicated on the plot.

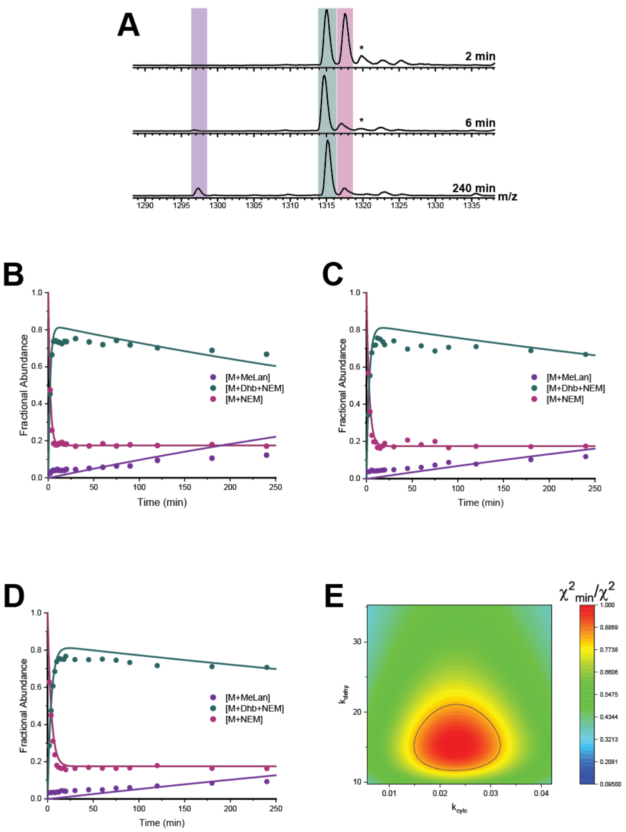

Figure S10. ProcA3.3 A' ring kinetics. A) Representative ESI mass spectra of the 8+ charge state at multiple time points. For each trace, the y-axis is scaled to the intensity of the highest peak present. The sample was treated with NEM to interrogate the cyclization state of the Cys residue. Species observed include unmodified (pink), dehydrated (teal), and cyclized (purple) peptide. This data was unable to be modeled due to the lack of kinetically competent cyclization. **B)** Time course for reaction containing 40 µM peptide and 2 µM ProcM. **C)** Time course for reaction containing 60 µM peptide and 2 µM ProcM. **D)** Time course for reaction containing 80 µM peptide and 2 µM ProcM. **E)** Confidence contour for the cyclization of the ProcA3.3 A' ring in isolation. The fit of the rate constants yielded a χ_{min}^2 of 1231 (χ^2/DOF = 8.2). The kinetic data shown here were simulated using the kinetic model shown in Scheme 2A of the main text. The confidence contours indicate the $\chi_{min}^2/\ \chi^2$ value as a function of variable parameters indicated on the x and y axis. Values with a $\chi_{min}^2/\ \chi^2$ value greater than 0.833 are within the black line indicated on the plot. Peaks labeled with an asterisk (*) are oxidation products that were present in the starting peptide and that were carried through to oxidized products.

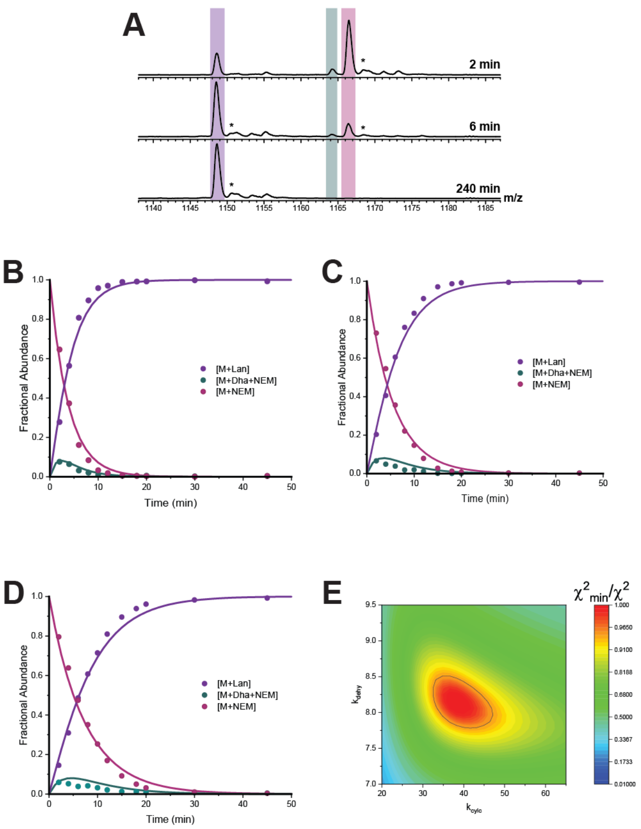

Figure S11. ProcA3.3 Variant Z A ring kinetics. A) Representative ESI mass spectra of the 8+ charge state at multiple time points. For each trace, the y-axis is scaled to the intensity of the highest peak present. The sample was treated with NEM to interrogate the cyclization state of the Cys residue. Species observed include unmodified (pink), dehydrated (teal), and cyclized (purple) peptide. **B)** Time course for reaction containing 40 μM peptide and 2 μM ProcM. **C)** Time course for reaction containing 60 μM peptide and 2 μM ProcM. **D)** Time course for reaction containing 80 μM peptide and 2 μM ProcM. **E)** Confidence contour for the cyclization of the ProcA3.3 variant Z A ring in isolation was calculated with rate constants listed in Table 2 of main text. The kinetic data shown here were simulated using the kinetic model shown in Scheme 2A of the main text. The fit of the rate constants listed in Table 2 yielded a χ_{min}^2 of 787.2 (χ^2/DOF = 5.7). The confidence contours indicate the χ_{min}^2/ χ^2 value as a function of variable parameters indicated on the x and y axis. Values with a χ_{min}^2/ χ^2 value greater than 0.96 are within the black line indicated on the plot. Peaks labeled with an asterisk (*) are oxidation products that were present in the starting peptide and that were carried through to oxidized products.

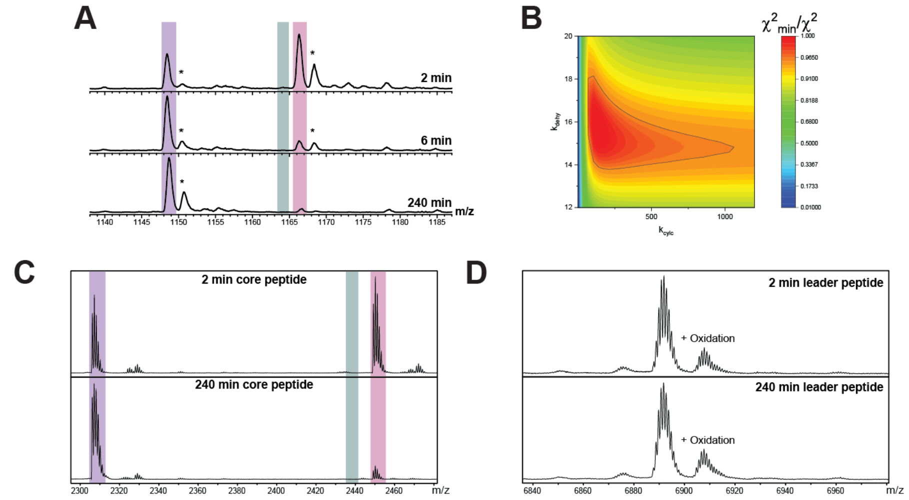

Species	Observed mass	Expected mass
[M+NEM+H]	2449.172	2449.130
[M-H_2O+H]	2306.114	2306.175

Species	Observed mass	Expected mass
[Leader peptide+H]	6887.417	6887.733
[Leader peptide+O+H]	6903.441	6903.463

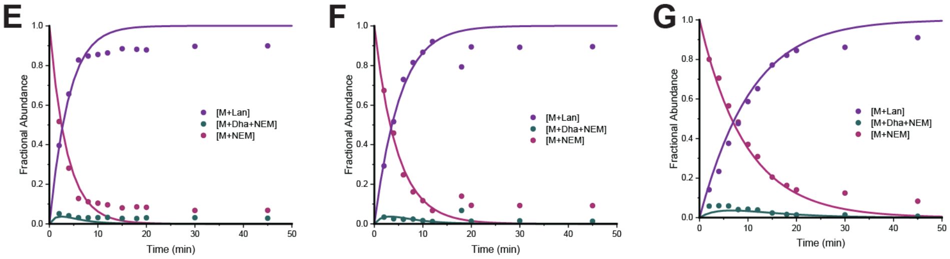

Figure S12. ProcA3.3 Variant Z A' ring kinetics. A) Representative ESI mass spectra of the 8+ charge state at multiple time points. For each trace, the y-axis is scaled to the intensity of the highest peak present. The sample was treated with NEM to interrogate the cyclization state of the Cys residue. Species observed include unmodified (pink), dehydrated (teal), and cyclized (purple) peptide. Peaks labeled with an asterisk (*) are oxidation products that were present in the starting peptide and that were carried through to oxidized products. The oxidation products are coming from the leader peptide (panels C/D). **B)** Confidence contour for the cyclization of the ProcA3.3 variant Z A' ring in isolation was calculated with rate constants listed in Table 2 of main text. The kinetic data shown here were simulated using the kinetic model shown in Scheme 2A of the main text. The fit of the rate constants listed in Table 2 yielded a χ_{min}^2 of 1779.6 (χ^2/DOF = 12.3). The confidence contours indicate the χ_{min}^2/χ^2 value as a function of variable parameters indicated on the x and y axis. Values with a χ_{min}^2/χ^2 value greater than 0.96 are within the black line indicated on the plot. **C)** MALDI-ToF mass spectra of $LahT_{150}$-digested ProcA3.3 Variant Z A' showing the core peptide intermediates. **D)** MALDI-ToF mass spectra of $LahT_{150}$-digested ProcA3.3 Variant Z A' showing the leader peptide. The observed oxidations in panel A are present on the leader peptide portion. **E)** Time course for reaction containing 60 μM peptide and 2 μM ProcM. **F)** Time course for reaction containing 80 μM peptide and 2 μM ProcM. **G)** Time course for reaction containing 80 μM peptide and 1 μM ProcM.

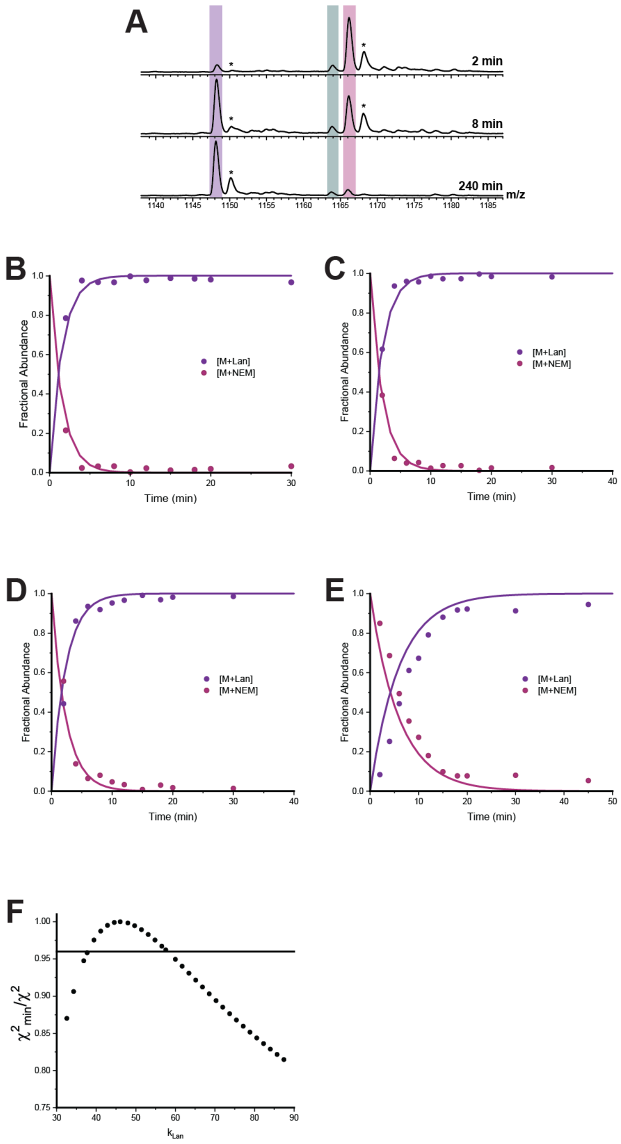

Figure S13. ProcA3.3 Variant Z B' ring kinetics. A) Representative ESI mass spectra of the 8+ charge state at multiple time points. For each trace, the y-axis is scaled to the intensity of the highest peak present. The sample was treated with NEM to interrogate the cyclization state of the Cys residue. Species observed include unmodified (pink) and cyclized (purple) peptide. **B)** Time course for reaction containing 40 μM peptide and 2 μM ProcM. **C)** Time course for reaction containing 60 μM peptide and 2 μM ProcM. **D)** Time course for reaction containing 80 μM peptide and 2 μM ProcM. **E)** Time course for reaction containing 80 μM peptide and 1 μM ProcM. **F)** One-dimensional confidence contour for the cyclization of the ProcA3.3 variant Z B' ring in isolation was calculated with the k_{lan} rate constant listed in Table 2 of the main text. The kinetic data shown here were simulated using the kinetic model shown in Scheme 2B of the main text. The fit of the rate constants listed in Table 2 yielded a χ_{min}^2 of 1944.5 (χ^2/DOF = 5.4). The confidence contours indicate the χ_{min}^2/χ^2 value as a function of k_{lan}. Values with a χ_{min}^2/χ^2 value greater than 0.96 are above the black line indicated on the plot. Peaks labeled with an asterisk (*) are oxidation products that were present in the starting peptide and that were carried through to oxidized products.

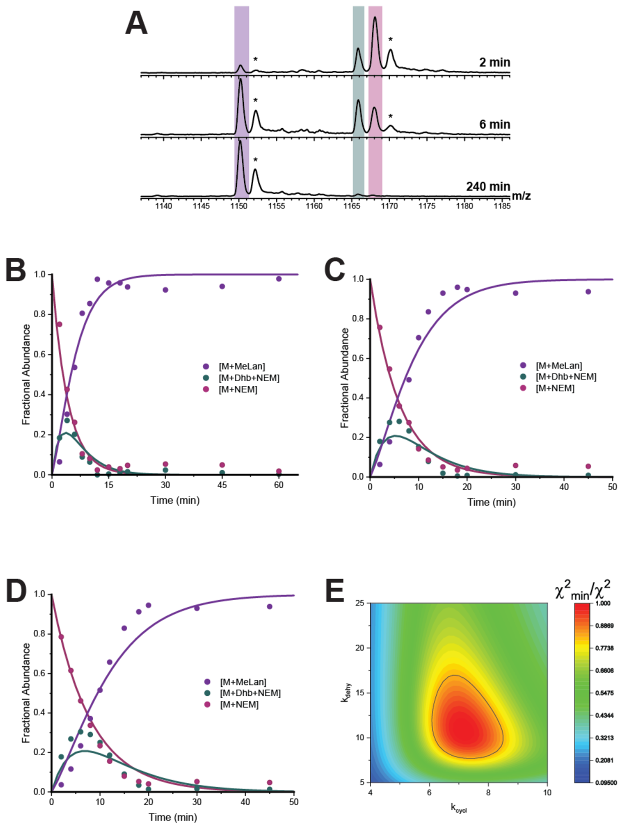

Figure S14. ProcA3.3 Variant Z MeLan A ring kinetics. A) Representative ESI mass spectra of the 8+ charge state at multiple time points. For each trace, the y-axis is scaled to the intensity of the highest peak present. The sample was treated with NEM to interrogate the cyclization state of the Cys residue. Species observed include unmodified (pink), dehydrated (teal), and cyclized (purple) peptide. **B)** Time course for reaction containing 40 µM peptide and 2 µM ProcM. **C)** Time course for reaction containing 60 µM peptide and 2 µM ProcM. **D)** Time course for reaction containing 80 µM peptide and 2 µM ProcM. **E)** Confidence contour for the cyclization of the ProcA3.3 variant Z MeLan A ring in isolation was calculated with rate constants listed in Table 2 of main text. The kinetic data shown here were simulated using the kinetic model shown in Scheme 2A of the main text. The fit of the rate constants listed in Table 2 yielded a χ_{min}^2 of 325.9 (χ^2/DOF = 2.2). The confidence contours indicate the $\chi_{min}^2/\ \chi^2$ value as a function of variable parameters indicated on the x and y axis. Values with a $\chi_{min}^2/\ \chi^2$ value greater than 0.83 are within the black line indicated on the plot. Peaks labeled with an asterisk (*) are oxidation products that were present in the starting peptide and that were carried through to oxidized products.

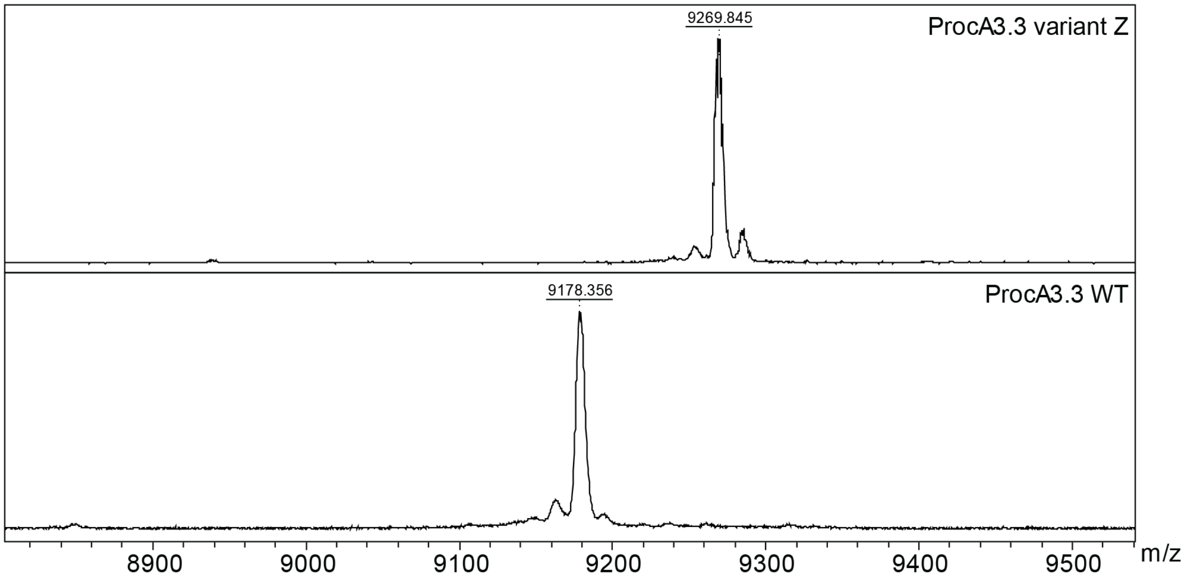

Figure S15. MALDI-ToF mass spectra for parent ProcA3.3 peptides. MALDI-ToF mass spectra were taken after peptide purification, thrombin digestion, and HPLC purification of the peptides.

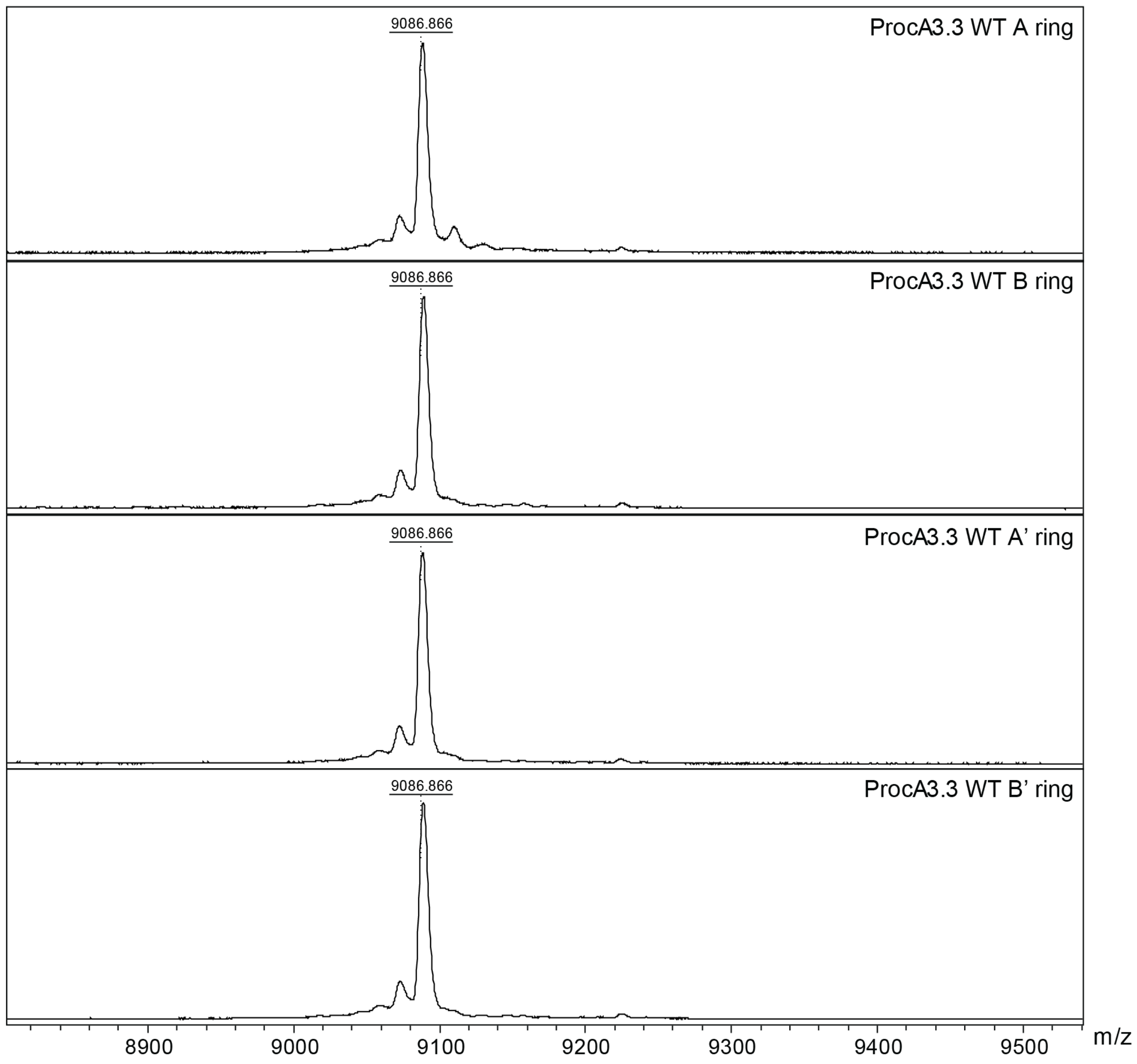

Figure S16. MALDI-ToF mass spectra for peptides used for the ProcA3.3 WT scaffold isolated ring analysis. MALDI-ToF mass spectra were taken after peptide purification, thrombin digestion, and HPLC purification of both peptides.

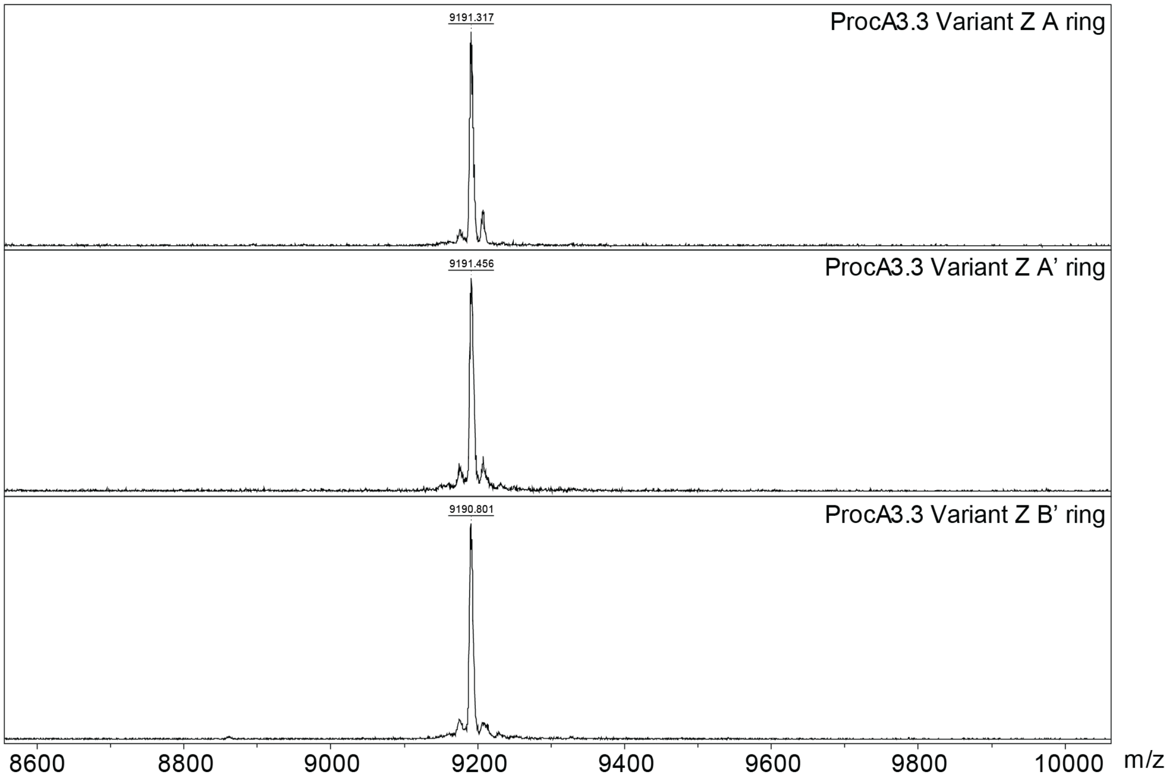

Figure S17. MALDI-ToF mass spectra for peptides used for the ProcA3.3 variant Z scaffold isolated ring analysis. MALDI-ToF mass spectra were taken after peptide purification, thrombin digestion, and HPLC purification of both peptides.

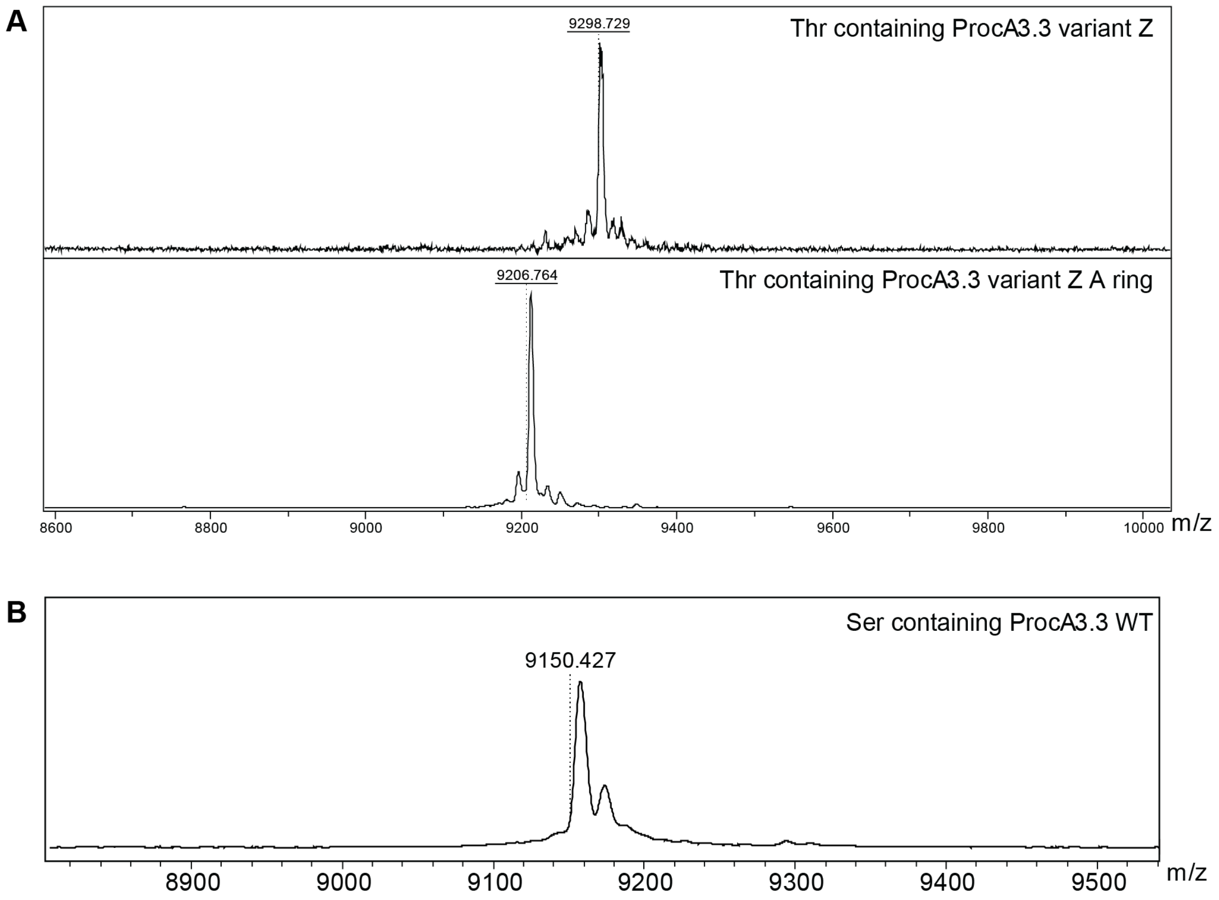

Figure S18. MALDI-ToF mass spectra for peptides used for Lan vs MeLan analysis. A) Thr containing ProcA3.3 variant Z peptides. **B)** Ser containing ProcA3.3 WT peptide. MALDI-ToF mass spectra were taken after peptide purification, thrombin digestion, and HPLC purification of both peptides.

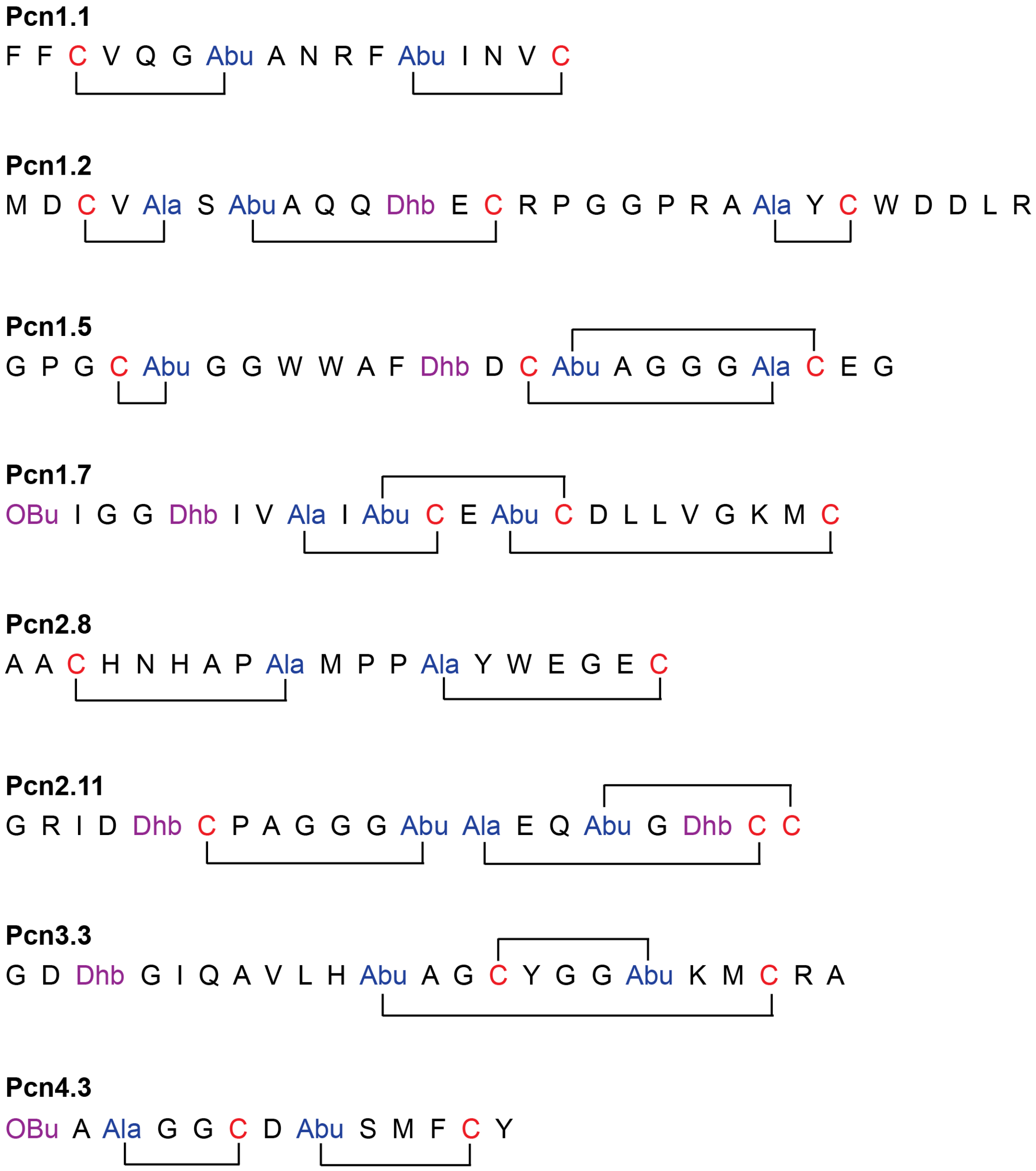

Figure S19. Ring patterns of various structurally characterized prochlorosins.

Table S1. Peak assignments for the peptide ions observed in thrombin digested substrates for the indicated ProcM-catalyzed reaction. Observed masses were determined using deconvoluted spectra.

Ion Species	Calculated Mass	Observed Mass	Error (ppm)	Reaction conditions, time point
**ProcA3.3 WT**				
M + 2 NEM + H	9428.5731	9428.5910	-1.9	60 µM, T=20
M + Dhb + 2 NEM + H	9410.5626	9410.5655	-0.3	60 µM, T=20
M + 2 Dhb + 2 NEM + H	9392.5521	9392.5680	-1.7	60 µM, T=20
M + MeLan + NEM + H	9285.5149	9285.5803	-7.0	60 µM, T=20
M + Dhb + MeLan + NEM + H	9267.5044	9267.5683	-6.9	60 µM, T=20
M + 2 Dhb + MeLan + NEM + H	9249.4939	9249.5713	-8.4	60 µM, T=20
M + 2 MeLan + H	9142.4567	9142.5104	-5.9	60 µM, T=20
M + 2 MeLan + Dhb + H	9124.4462	9124.5261	-8.8	60 µM, T=20
**ProcA3.3 variant Z**				
M + 2 NEM + H	9520.59594	9520.5427	5.6	40 µM, T=15
M + Dha + 2 NEM + H	9502.58544	9502.5789	0.7	40 µM, T=15
M + Lan + NEM + H	9377.53774	9377.4791	6.3	40 µM, T=15
M + Dha + Lan + NEM + H	9359.52724	9359.4927	3.7	40 µM, T=15
M + 2 Dha + Lan + NEM + H	9341.51674	9341.4790	-4.0	40 µM, T=15
M + 2 Lan + H	9234.47954	9234.5075	-3.0	40 µM, T=8
M + 2 Lan + Dha + H	9216.46904	9216.4979	-3.1	40 µM, T=8
**ProcA3.3 A ring**				
M + Dhb + NEM + H	9211.5322	9211.6028	-7.7	40 µM, T=2
M + NEM + H	9193.5217	9193.5620	-4.4	40 µM, T=2
M + Lan+ H	9068.474	9068.5443	-7.8	40 µM, T=2
**ProcA3.3 variant Z A ring**				
M + Dha + NEM + H	9317.5707	9317.6279	-6.1	40 µM, T=2
M + NEM + H	9299.5602	9299.5924	-3.5	40 µM, T=2
M + Lan+ H	9174.5125	9174.5656	-5.8	40 µM, T=2
**ProcA3.3 variant Z MeLan A ring**				
M + Dhb + NEM + H	9331.5863	9331.6016	-1.6	40 µM, T=4
M + NEM + H	9313.5758	9313.6098	-3.6	40 µM, T=4
M + Lan+ H	9188.5281	9188.6069	-8.6	40 µM, T=4

References

(1) Bobeica, S. C.; Dong, S. H.; Huo, L.; Mazo, N.; McLaughlin, M. I.; Jimenéz-Osés, G.; Nair, S. K.; van der Donk, W. A. Insights into AMS/PCAT transporters from biochemical and structural characterization of a double glycine motif protease. *eLife* **2019**, *8*, e42305.

(2) Brademan, D. R.; Riley, N. M.; Kwiecien, N. W.; Coon, J. J. Interactive peptide spectral annotator: A versatile web-based tool for proteomic applications. *Mol. Cell. Proteom.* **2019**, *18*, S193-S201.
